# Indolyl Septanoside Synthesis for In Vivo Screening of Bacterial Septanoside Hydrolases

**DOI:** 10.3390/ijms22094497

**Published:** 2021-04-26

**Authors:** Aditya R. Pote, Sergi Pascual, Antoni Planas, Mark W. Peczuh

**Affiliations:** 1 Department of Chemistry, University of Connecticut, 55 N. Eagleville Road U3060, Storrs, CT 06269, USA; aditya.pote@gmail.com; 2 Laboratory of Biochemistry, Institute Químic de Sarrià, University Ramon Llull, 08017 Barcelona, Spain; sergipascualt@iqs.url.edu

**Keywords:** septanoside, indolyl glycoside, glycosidase, septanoside hydrolase

## Abstract

Building-up and breaking-down of carbohydrates are processes common to all forms of life. Glycoside hydrolases are a broad class of enzymes that play a central role in the cleavage of glycosidic bonds, which is fundamental to carbohydrate degradation. The large majority of substrates are five- and six-membered ring glycosides. Our interest in seven-membered ring septanose sugars has inspired the development of a way to search for septanoside hydrolase activity. Described here is a strategy for the discovery of septanoside hydrolases that uses synthetic indolyl septanosides as chromogenic substrates. Access to these tool compounds was enabled by a route where septanosyl halides act as glycosyl donors for the synthesis of the indolyl septanosides. The screening strategy leverages the known dimerization of 3-hydroxy-indoles to make colored dyes, as occurs when the β-galactosidase substrate X-Gal is hydrolyzed. Because screens in bacterial cells would enable searches in organisms that utilize heptoses or from metagenomics libraries, we also demonstrate that septanosides are capable of entering *E. coli* cells through the use of a BODIPY-labeled septanoside. The modularity of the indolyl septanoside synthesis should allow the screening of a variety of substrates that mimic natural structures via this general approach.

## 1. Introduction

**Figure 1:**
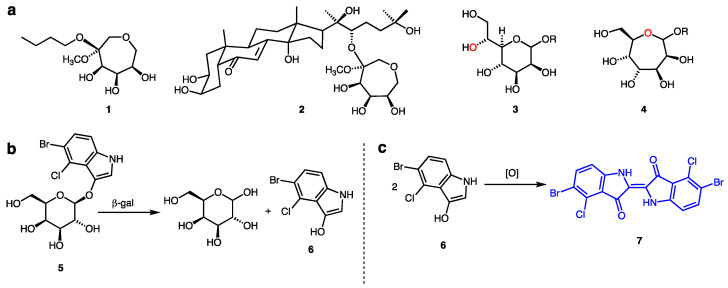
(**a**) Portulasoid **1** and 20-hydroxy-ecdysone septanoside **2** from *Atriplex portulacoides* roots; d-*glycero*-d-*manno*-heptose in its pyranose (**3**) and septanose (**4**) ring forms. (**b**) Hydrolysis of X-Gal **5**, giving rise to d-galactose and 3-hydroxyindole **6**. (**c**) Oxidative dimerization of 3-hydroxyindole **6** to indigo dye **7**.

Glycoside hydrolases (GH) are a broad superfamily of carbohydrate active enzymes that perform important functions in glycobiology—degradation of structural polysaccharides, remodeling of cell-surface proteoglycans and glycolipids, and even activation of small molecule natural products from latent precursor compounds [1,2,3,4,5]. Glycoside hydrolases (also referred to as glycosidases) cleave acetal linkages (glycosides) between a sugar and an aglycone moiety. Classification of GH families is based on the configuration (α- vs. β-) and location of the hydrolyzed bond (i.e., *endo-* vs. *exo-*), the identity of the sugar residue that is cleaved (e.g., d-glucose and d-galactose), and the three-dimensional shape of the hydrolase. The widely used repository of Carbohydrate Active Enzymes (CAZy database, www.cazy.org (accessed on 1 March 2021)) [6] classifies CAZymes in families based on sequence similarity and three-dimensional (3D) folds. Among glycoside hydrolases, there are currently (March 2021) 170 GH families. Members of each family share the same reaction mechanism and 3D fold, but different substrate specificities are found in most of the families, meaning that substrate specificity is dictated by subtle structural differences at the active site. Substrates typically consist of a pyranose or a furanose ring attached to a variety of aglycone species. Seven-membered ring septanose glycosides are all but unprecedented in natural systems [7,8]. While they are not true septanosides because the seven-membered ring is not linked through a glycosidic linkage, the recently reported natural products portulasoid **1** and 20-hydroxy-ecdysone septanoside **2** from *Atriplex portulacoides* roots (Figure 1a) [9] open the provocative possibility that other similar structures, in which the polyhydroxy oxepane might be a septanoside (e.g., **4** in Figure 1a), could be discovered. In principle, it might be possible that septanoside hydrolases are present in nature or that promiscuous glycoside hydrolases might also accept septanoside substrates. Indeed, it is known that the seven-member ring iminosugar 1,6-dideoxy-1,6-imino-l-iditol was a glycosidase inhibitor [10], and it was reported that 4-nitrophenyl l-idoseptanosides, septanose analogs from hexoses, were substrates of some glycoside hydrolases, albeit weak ones [11].

The identification of a naturally occurring septanoside hydrolase or other GH sufficiently promiscuous to hydrolyze septanosides would be an important milestone. Such an enzyme, or mutants thereof, would open up whole new areas of glycobiology research. For example, discovery of a septanoside hydrolase would motivate the search for septanoside-containing glycoconjugates in the producing organism. Plants are a candidate kingdom in this search because of the discovery of compounds **1** and **2**. An even more promising source, however, might be Gram-negative bacteria. There are numerous examples of heptose-containing glycoconjugates present in Gram-negative species [12,13], with the sedoheptulose/d-*glycero*-d-*manno*-heptose (**3**) biosynthetic pathway being a representative [14]. Cyclization of **3** through its C6 hydroxyl group (red) instead of the C5 hydroxyl could ultimately give rise to septanose glycosides **4**. Biosynthetic pathways involving heptoses are still in the early days of being thoroughly characterized, which also adds an incentive to a search for new glycosyl hydrolases. A first step in any search for septanoside hydrolases, therefore, is the development of tools—an assay strategy and matching substrate—that could be used to identify these enzymes.

One straightforward approach to search for septanoside hydrolases borrows from the β-galactosidase assay widely used in molecular biology. Upon glycoside hydrolysis of a pro-chromogenic substrate such as 5-bromo-4-chloro-3-indolyl-β-d-galactopyranoside **5** (X-Gal) [15], d-galactose is liberated along with 3-hydroxyindole **6** (Figure 1b). Oxidative dimerization of **6** results in the formation of an easily detected indigo dye **7**. Development of a blue color in a bacterial colony or culture of when X-Gal is present therefore reports on the presence of the β-galactosidase enzyme. In practice, the “blue-white colony screen” is frequently applied to the preparation of a protein expression vector [16]. The multiple cloning site where a DNA construct encoding the protein of interest will be inserted is positioned within the β-galactosidase gene on the plasmid. Plasmids lacking the insert retain a functioning β-galactosidase and hence lead to blue colonies after transformation. Successful insertion of the construct into the plasmid, on the other hand, disrupts the β-galactosidase gene and the associated hydrolase activity, giving rise to white colonies after transformation. In the context of our search, we envisioned using indolyl septanosides as candidate substrates of putative septanoside hydrolases. If hydrolytic activity toward such a substrate were present in a bacterial colony, we would anticipate the appearance of a characteristic blue color. To that end, we report here the syntheses of indolyl septanoside substrates for the discovery of septanoside hydrolases. Their structures mimic X-Gal, allowing them to be used for in vivo screening of bacteria. We further demonstrate that a related BODIPY–septanoside conjugate is transported into the cytoplasm of *E. coli*, probably using any of its numerous sugar transporters. Development of these tools constitutes the first steps toward the identification of septanoside hydrolase enzymes.

## 2. Results

### 2.1. Synthesis of Indolyl Septanosides

**Figure 2:**
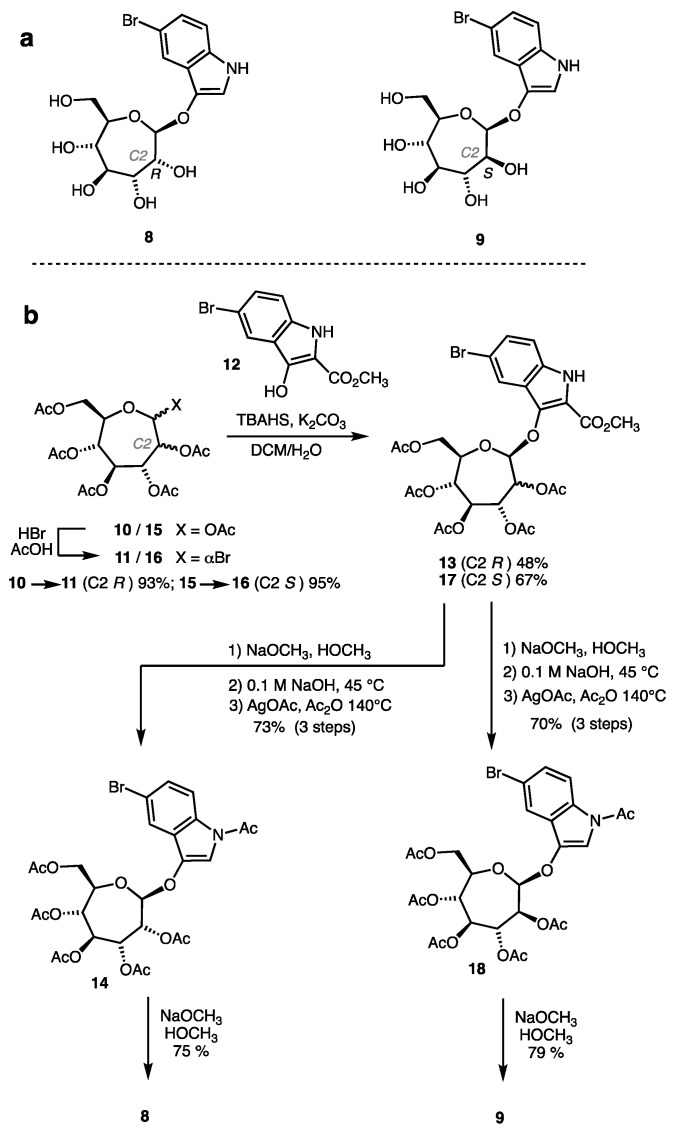
(**a**) Indolyl septanosides **8** and **9**. (**b**) Synthetic routes, including yields, used to prepare indolyl septanosides **8** and **9**.

Indolyl septanosides **8** and **9** (Figure 2a) became the initial targets of substrate synthesis for a few reasons. First among them were the structural analogy of **8** and **9** to X-Gal **5** and reports on methods for the synthesis and utilization of indolyl glycosides in glycosidase assays [17]. The substitution pattern on the 5-bromo-3-indolyl aglycone was based on the ready availability of the 5-bromo-anthranilic acid starting material and the color of tyrian purple dye (an analog of **7** lacking the chlorine atoms) that arises from its oxidative dimerization [18,19]. Further, the stereogenic centers from C3-C6 in β-configured d-*glycero*-d-*gulo*-septanoside **8** and d-*glycero*-d-*ido*-septanoside **9** have identical relative configurations as D-glucose from C2-C5. The “phased” correspondence between these centers was important to recognition of septanosides by lectins [20,21,22]; it refers to the fact that C2 of the pyranose is the same as the C3 of the septanose and so forth. We also considered the possible promiscuity of glucosidases as giving us a higher probability of identifying active GHs in our search. The synthetic strategy drew from reported methods for indolyl glycoside synthesis [18,23,24,25] and our previous experience at preparing septanose glycosides via nucleophilic displacement on anomeric bromides [26]. It leveraged a synthesis of the per-*O*-acetyl septanose precursors of the anomeric bromides that began from natural D-pyranosides such as D-glucose, suggesting that the route could be extended to other sugars.

Preparation of indolyl septanoside **8** leveraged the known conversion of per-*O*-acetyl septanose **10** to its corresponding α-configured anomeric bromide **11** (93%, Figure 2b) [26]. Attempted glycosylation of **11** via direct displacement (S_N_2) conditions with 5-bromo-indoxyl (i.e., the analog of **12** lacking the C2 carboxylate group) was unsuccessful due to rapid oxidative dimerization of the indoxyl species under the basic reaction conditions (**11** + **12**, potassium *tert*-butoxide in acetonitrile, 0 °C to rt). We therefore resorted to a strategy that used 2-carbomethoxy indoxyl **12** as acceptor. The carbomethoxy moiety at the 2-position of the indoxyl ring is a blocking group that prevents oxidative dimerization during the glycosylation [18]. In the event, glycosylation of **11** with **12** gave protected septanoside **13** in 48% yield; the yield for the glycosylation is modest but was not extensively optimized. Recompense for successful glycosylation in this instance was the multi-step deprotection that had to be undertaken to arrive at the target. Compound **13** was therefore converted to key intermediate **14** by a sequence that included removal of the acetate protecting groups and the methyl esters in a two-step process. Re-acetylation of the hydroxyl groups in this species was concomitant with decarboxylation of the carboxy group yielding **13** in 73% over the three steps. It proved convenient to purify **13** at this stage before the final de-acetylation. Removal of the acetates under Zemplén conditions then provided 5-bromo-3-indolyl septanoside **8** in 75% yield. Overall, the yield of **8** over the six-step sequence was 24% with an average yield per step of 73%. Compound **9**, the C2′-epimer of **8**, was prepared from per-*O*-acetyl septanose **15** using the same set of reactions, and shown in Figure 2b, in 35% yield over the six-step sequence.

Indolyl septanoside **8** was characterized both structurally and functionally. NMR spectroscopic data for **8** were consistent with a β-configured anomeric center (H1 δ = 4.93 ppm, C1 δ = 110.1 ppm) and a trans disposition of the groups at C1 and C2 (^3^*J*_H1,H2_ = 5.5 Hz). These values were comparable to other 1,2-trans β-septanosides we have synthesized, including *p*-nitrophenyl (pNP) septanoside **19** (vide infra). Values for the 1,2-cis indolyl septanoside **9** (H1 δ = 5.09 ppm, C1 δ = 105.5 ppm, and ^3^*J*_H1,H2_ = 2.3 Hz) were also consistent with its proposed structure [8,26]. In a separate line of experimentation, septanoside **9** was exposed to buffer solutions at pH 5, 6, and 7, as well as a 1 M solution of HCl. With standing at room temperature over 8 h, color developed only in the 1 M HCl samples. We interpreted the results to be a demonstration of the stability of the indolyl glycosides at pH values near neutrality and in a range where many GH enzymes are active. The results also demonstrated the susceptibility to acid hydrolysis. In a preparative scale experiment, the indigo precipitate that arose from 1M HCl hydrolysis of **9** was collected, redissolved in *N*,*N*-dimethylformamide, and its UV–vis spectrum compared to a sample of tyrian purple synthesized independently by a different method [27,28,29].

### 2.2. Evaluation of Indolyl Septanoside ***8*** as a Substrate of Common Exo-Glycoside Hydrolases

**Figure 3:**
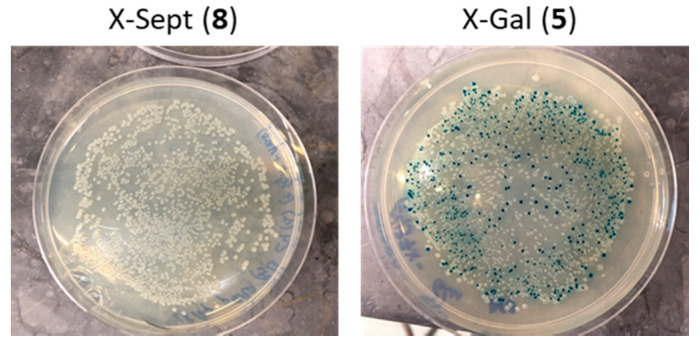
Blue-white colony screen of *E. coli* BL21(DE3) Star cells with indolyl septanoside **8**. BL21(DE3) Star cells harboring a plasmid expressing *E. coli* β-galactosidase: (**Left**) with X-Sept (**8**) substrate; and (**Right**) with substrate X-Gal (**5**).

Compound **8** was assayed in vivo with *E. coli* cells under conditions that are typical for assays that use X-Gal substrate **5**. *E. coli* BL21(DE3) Star cells harboring an inducible plasmid overexpressing the *E. coli* β-galactosidase (BL21-pGal^+^) and cell harboring an empty plasmid (BL21-pGal^−^) were grown on Petri plates containing compound **8** with isopropyl 1-thio-β-d-galactopyranoside (IPTG) as inducer. No blue colonies were observed (as opposed to the control BL21-pGal^+^ with **7**) (Figure 3). At first glance, the apparent lack of activity, as evidenced by the lack of appearance of blue colonies, may reflect that *E. coli* β-galactosidase did not recognize septanoside **8**. It may also have indicated that the substrate was not internalized into the *E. coli* cells. To test the first scenario, we assayed the purified *E. coli* β-galactosidase with compound **8** under the same experimental conditions where it shows maximum activity with its cognate substrate (Table 1). Once again, no hydrolytic activity was detected, confirming that septanoside **8** is not a β-galactosidase substrate.

**Figure 4:**
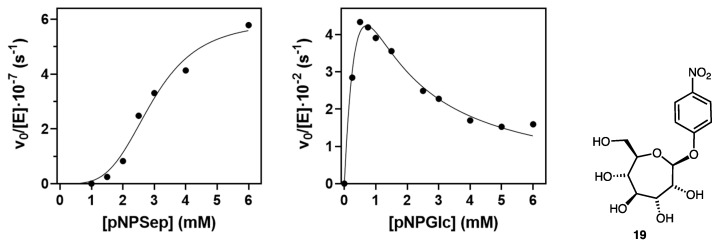
Kinetics of *Streptomyces* sp. β-galactosidase (Bgl3) with pNP-Sept (**19**) and pNP-Glc substrates. Conditions: 50 mM phosphate buffer, pH 6.5, and 50 °C.

With the aim of potentially finding an enzyme that was able to recognize septanoside substrates, indolyl septanoside **8** was subjected to a panel of glycosidases to test in vitro for their ability to hydrolyze the substrate (Table 1). Each enzyme was assayed at high enzyme concentration (25–50 U/mL, activity units with its cognate substrate) with compounds **8** and **9** (1 mM) at the pH optimum of each enzyme. None of them showed measurable activity (by colorimetry of released indigo dye), including the *E. coli* β-galactosidase as indicated above. Only the *Streptomyces* sp. β-glucosidase (Bgl3) resulted in faint activity. This is a rather promiscuous enzyme reported to hydrolyze aryl glycosides of d-glucose, d-galactose, d-mannose, d-xylose and l-fucose [30]. We then analyzed the activity of Bgl3 against *p*-nitrophenyl septanoside **19**, prepared as previously reported [26], in greater detail. At a fixed Bgl3 concentration (9.2 μM), initial rates at increasing concentrations of **19** (Figure 4) were fit to give the following kinetics parameters: *k_cat_* = 5.9 × 10^−7^ min^−1^; *K_M_* = 3 mM; *h* = 3.8. As compared to an efficient Bgl3 substrate (pNP-β-d-glucopyranoside, *k_cat_* = 13.9 × 10^−2^ min^−1^, *K_M_* = 0.8 mM, *K_i_* = 0.6 mM), the data show that **19** is a poor substrate for the glycosidase with a turnover of approximately five orders of magnitude slower than the glucoside.

### 2.3. Transport of a BODIPY-Labeled Septanoside into E. coli Cells

**Figure 5:**
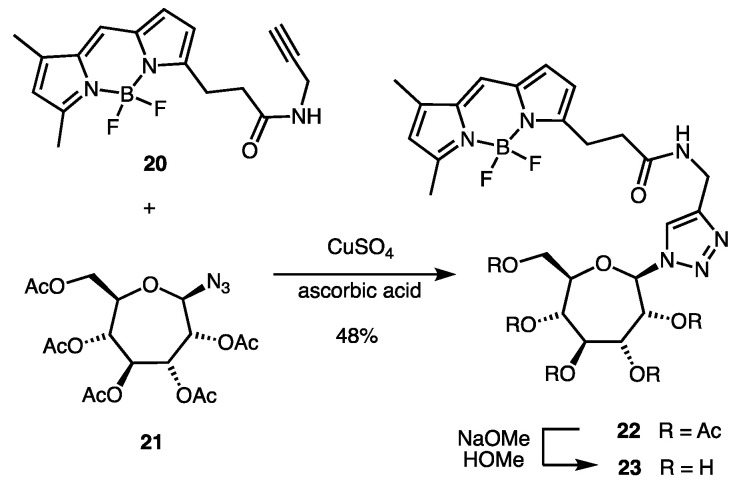
Synthesis of BODIPY-labeled septanoside **23**.

In search of septanoside hydrolases, we intend to use compound **8** for the screening of plant, yeast, and bacterial cell extracts as well as for in vivo “blue-white” screening of bacterial cells harboring libraries of glycoside hydrolases (i.e., metagenomics libraries) [31]. It was necessary, therefore, to return to the question of whether or not **8** was capable of entry into *E. coli* cells. To that end, we investigated the transport of septanosides into the bacterial cytoplasm. As a proof-of-concept, a BODIPY-labeled septanoside (i.e., **23**, Figure 5) was synthesized as a surrogate of compound **8** to be used in confocal microscopy studies. BODIPY is a commonly used fluorophore tag that can be used to analyze uptake by cells [32,33] and for cell sorting by fluorescence-activated cell sorting (FACS) [34,35]. A Huisgen click reaction [33,36] between commercially available BODIPY alkyne **20** and the known septanosyl azide **21** [26] gave protected conjugate **22** in 48% yield. Deprotection of compound **22** under Zemplén conditions then afforded BODIPY-labeled septanoside **23**.

**Figure 6:**
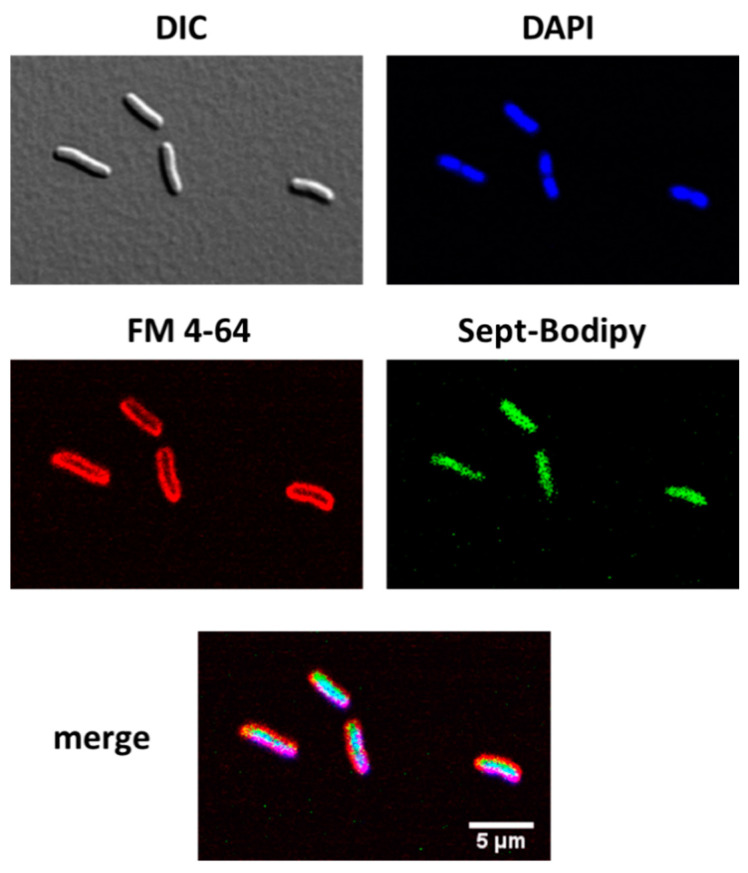
Confocal fluorescence microscopy of *E. coli* MG1655 cells with labeling stains. DIC, optical microscopy image; DAPI, DNA staining (bacterial chromosome); FM 4-64, membrane staining; Sept-BODIPY, BODIPY-labeled septanoside (**23**). Scale bar (5 μm) is the same in all images.

Two different *E. coli* strains, MG1655 and TOP10, were used to analyze cellular uptake by confocal microscopy (Figure 6). Prior to microscopy, cultures were grown to OD 1.0, washed, and then suspended in 10 mM MES buffer at pH 5.5. An aliquot of this culture was incubated with FM4-64 (localizes into bacterial membrane), BODIPY septanoside **23**, and DAPI (DNA stain), and then spun down and resuspended prior to observation. With MG1655 cells, the BODIPY fluorescence clearly localized in the cytoplasm with high intensity, confirming internalization of the septanoside. A lack of BODIPY fluorescence intensity at the cell periphery indicated that the labeled septanoside was not attached to the outer membrane. The merged image in Figure 6 shows the colocalization of the blue and green images, consistent with transport of **23**, which was considered a surrogate for indolyl septanoside **8** in these experiments. Similar results were obtained in experiments with the TOP10 cells, where **23** also localized in the cytoplasm of this *E. coli* strain (see Supplementary Materials).

## 3. Discussion

The investigation reported here has established a conceptual framework behind the search for septanoside hydrolases from natural sources. Such a search will allow the identification of catabolic enzymes from organisms such as *Atriplex portulacoides* that produce polyhydroxy oxepanes (the source of compounds **1** and **2**) or Gram-negative organisms that utilize heptoses. Additionally, metagenomics libraries expressed in bacteria such as *E. coli* can be interrogated as well for hydrolytic activity. Our approach is fundamentally molecular—small molecule synthesis was used to prepare tools that enable searching both in vitro and, critically, in vivo for enzymes. Indolyl septanosides are useful reagents because they enable the colorimetric characterization of glycoside hydrolysis in a manner analogous to X-Gal. Preliminary assays in *E. coli* to characterize the reactivity of indolyl d-*glycero*-d-*gulo*-septanoside **8** as a substrate of β-galactosidase showed no hydrolysis. The apparent lack of reactivity of this septanoside substrate was subsequently explored along two different lines. First, in vitro experiments with a panel of commercially available glycosidases also showed no significant hydrolysis of the unnatural substrates **8** and **9**. Significantly, Bgl3 (β-glucosidase from *Streptomyces* sp.) showed a suggestive activity with light-pale blue colonies when using substrate **8**. Further analysis with *p*-nitrophenyl septanoside **19** confirmed the low activity, in the range of 5–6 orders of magnitude lower than its *p*-nitrophenyl D-glucoside substrate. In a second line of inquiry, BODIPY-labeled septanoside **23**, prepared via click reaction with a septanosyl azide, was observed inside *E. coli* cells by confocal microscopy. It suggested that septanosides traverse the bacterial envelope. Taken together, these results illustrate an approach that opens the door to a broader search for septanoside hydrolase activity in bacterial systems.

## 4. Materials and Methods

### 4.1. General Experimental

Commercially available starting materials, reagents, and solvents (Sigma-Aldrich, St. Louis, MO, USA and Acros/Thermofisher, Waltham, MA, USA) were used without further purification. Septanosyl azide **21** was prepared as previously reported [26]. Reactions were performed under nitrogen atmosphere unless otherwise noted and were monitored by TLC using silica gel HL plates w/UV254, 250 μm (SiliCycle, Quebec City, PQ, Canada), visualized either under UV lamp or by charring with 2.5% *p*-anisaldehyde in H_2_SO_4_, AcOH and EtOH solutions. Reverse phase C18 TLC plates w/UV254, 200 μm (Sorbtech, Atlanta, GA, USA) were also used and visualized by UV. Flash chromatography was performed on silica gel (60 Å, 40–63 μm). ^1^HNMR spectra were collected on Bruker NMR instruments at either 400 and 500 MHz with chemical shift referenced to (CH_3_)_4_Si (δ_H_ 0.00 ppm) or the residual peak in CDCl_3_ (δ_H_ 7.24 ppm) or CD_3_OD (δ_H_ 3.31 ppm). ^13^C NMR spectra were collected at 100 MHz and referenced to residual peak in CDCl_3_ (δ_C_ 77.2 ppm) or CD_3_OD (δ_C_ 49.1 ppm). High resolution mass spectrometry data were collected on a JEOL DART Electrospray-Time-of-Flight (AccuTOF-DART) instrument. Enzymes were purchased from Megazyme Ltd. (Bray, Ireland). β-Glucosidase from *Streptomyces* was expressed and purified as reported in [30]. DAPI (4′,6-diamidine-2′-phenylindole dihydrochloride) and FM 4-64 (*N*-(3-triethylammoniumpropyl)-4-(6-(4-(tiethylamino)phenyl)hexatrienyl)pyridinium dibromide) were purchased from Sigma-Aldrich and Thermofisher, respectively.

### 4.2. Synthesis of Indolyl Septanosides ***8*** and ***9***

*Phase-Transfer Glycosylation (General Procedure).* Freshly prepared per-*O*-acetyl septanosyl bromide (i.e., **10**) [26] (0.306 g, 0.63 mmol), tetra-*N*-butylammonium hydrogen sulfate (TBAS, 0.214 g, 0.63 mmol), and 2-methoxycarbonyl-3-hydroxy-5-bromo-indole **11** (0.210 g, 0.79 mmol) were mixed in DCM (8 mL). To this solution was added 12 mL of a 1 M aqueous solution of K_2_CO_3_. The biphasic (organic:aqueous) reaction mixture was stirred vigorously at room temperature until consumption of the bromide was complete as determined by TLC (1:1 Hex:EtOAc). Afterwards, the organic phase was separated from the aqueous phase; the aqueous phase was extracted with additional DCM (1 × 12 mL). The combined organic layers were dried with Na_2_SO_4_, filtered, and the and the solvent was removed under reduced pressure. The residue was then purified by column chromatography in the solvent mixtures stated.

5-bromo-2-methoxycarbonyl-indol-3-yl 2,3,4,5,7-penta*-O-*acetyl-β-d-*glycero*-d-*gulo*-septanoside (**13**). Obtained as brownish oil from **11** and **12** to yield 0.210 g (48%) of **13**. R*_f_* 0.4 (1:1 Hex: EtOAc); ^1^H NMR (400 MHz, CDCl_3_) δ 9.28 (s, 1H), 7.93 (broad s, 1H), 7.36 (dd, *J* = 8.8, 1.8 Hz, 1H), 7.23 (d, *J* = 8.8 Hz, 1H), 5.74 (dd, *J =* 4.7, 2.2 Hz, 1H), 5.64 (dd, *J =* 8.8, 2.2 Hz, 1H), 5.49–5.45 (m, 1H), 5.30 (d, *J =* 4.8 Hz, 1H), 5.22 (dd, *J =* 9.4, 6.0 Hz, 1H), 4.13 (dd, *J =* 14.3, 7.1 Hz, 1H), 4.08–4.02 (m, 1H), 3.90 (s, 3H), 3.88–3.85 (m, 1H), 2.22 (s, 3H), 2.12 (s, 3H), 2.11 (s, 3H), 2.01 (s, 3H), 1.72 (s, 3H); ^13^C NMR (100 MHz, CDCl_3_) δ 170.6, 169.5, 169.4, 169.3, 169.1, 161.4, 139.0, 132.5, 129.2, 123.4, 123.2, 117.0, 113.8, 113.7, 105.4, 73.3, 72.7, 71.2, 70.9, 69.3, 63.5, 60.5, 52.1, 21.0, 20.7, 20.7(2), 20.3, 14.2; HRMS (DART-TOF) *m*/*z* calcd. for C_27_H_30_BrNO_12_Na [M+Na]^+^ 694.0747, obs. 694.0743.

5-bromo-2-methoxycarbonyl-indol-3-yl 2,3,4,5,7-penta-*O*-acetyl-β-d-*glycero*-d-*ido*-septanoside (**17).** Reaction of **12** with septanosyl bromide **16** using the general procedure gave 0.160 g (67%) of **17** as a brownish syrup. R*_f_* 0.3 (3:2 Hex: EtOAc) ^1^H NMR (400 MHz, CDCl_3_) δ 9.16 (s, 1H), 7.96 (d, J = 1.7 Hz, 1H), 7.37 (dd, J = 8.8, 1.8 Hz, 1H), 7.23 (d, J = 8.8 Hz, 1H), 5.66 (dd, J = 7.4, 2.1 Hz, 1H), 5.61 (m, 1H), 5.52 (d, J = 2.2 Hz, 1H), 5.40–5.33 (m, 2H), 4.09 (dd, J = 12.2, 5.7 Hz, 1H), 3.98 (s, 3H), 3.94 (dd, J = 12.0, 2.2 Hz, 1H), 3.92 (ddd, J = 11.0, 5.8, 2.2 Hz, 1H), 2.18 (s, 3H), 2.10 (s, 3H), 2.04 (s, 3H), 1.98 (s, 3H), 1.68 (s, 3H); ^13^C NMR (100 MHz, CDCl_3_) δ 170.8, 170.0, 169.5, 169.2, 169.0, 161.4, 139.6, 132.6, 129.5, 123.4, 123.1, 116.5, 114.0, 113.7, 103.2, 76.4, 72.1, 72.0, 70.5(2), 63.4, 60.6, 52.4, 20.9, 20.7, 20.6(2), 20.3, 14.3; HRMS (DART-TOF) m/z calcd. for C_27_H_31_BrNO_12_ [M+H]^+^ 672.0928, obs. 672.0923.

*Multistep De-acetylation, Decarboxylation, and Acetylation (General Procedure).* At room temperature, a solution of approximately 0.3 mmol of the protected indolyl septanoside (i.e., **13** or **17**) in 5 mL MeOH was treated with a catalytic amount of sodium methoxide (5 mol %). After starting material disappeared from the TLC (1:1 Hex:EtOAc), indicating that deacetylation was complete, the solvent was removed, and NaOH (0.1 M aq.; 14 mL) was added to the residue. The mixture was heated to 40–45 °C and stirred until C18 reverse-phase TLC indicated that ester hydrolysis was complete, approximately 4 h (R_f_ of the product is 0.7 and starting material is 0.3 in 3:1 water:acetonitrile). The mixture was lyophilized, then AgOAc (3.0 equiv.), K_2_CO_3_. (6.0 equiv.), and Ac_2_O (10.0 mL) were added. The mixture was heated to 110 °C for 1 h. Then the mixture was cooled to room temperature and diluted with 20 mL water and 20 mL CH_2_Cl_2_. The organic phase was washed with water (2 × 20 mL) and dilute aqueous NaHCO_3_ (~0.1 M, 1 × 20 mL). The organic phase was dried with Na_2_SO_4_, filtered, and the eluent was removed under reduced pressure. The residue was then purified by column chromatography in the solvent stated.

*N*-Acetyl-5-bromoindol-3-yl 2,3,4,5,7-penta-*O*-acetyl-β-d-*glycero*-d-*gulo*-septanoside (**14**). Obtained as a yellowish solid from **13** using the general procedure (73% yield over 3 steps). R*_f_* 0.3 (1:1 Hex:EtOAc) ^1^H NMR (400 MHz, CDCl_3_) δ 8.25 (broad s, 1H), 7.69 (d, *J =* 1.8 Hz, 1H), 7.46 (dd, *J =* 8.8, 1.8 Hz, 1H), 7.30 (broad s, 1H), 5.57–5.54 (m, 2H), 5.44 (dd, *J =* 8.0, 4.7 Hz, 1H), 5.15 (d, *J =* 5.4 Hz, 1H), 4.33–4.31 (m, 1H), 4.17–4.09 (m, 3H), 2.62 (s, 3H), 2.20 (s, 3H), 2.14 (s, 3H), 2.13 (s, 3H), 2.06 (s, 3H), 2.03 (s, 3H), 1.93 (s, 3H); ^13^C NMR (100 MHz, CDCl_3_) δ 170.6, 169.3(2), 169.2, 169.0, 168.5, 140.0, 132.3, 129.1, 126.1, 120.8, 118.1, 117.2, 112.2, 103.5, 74.0, 72.2, 71.6, 70.6, 69.2, 63.7, 60.5, 24.0, 21.2, 21.0, 20.8(2), 20.7, 14.3; HRMS (DART-TOF) *m/z* calcd. for C_27_H_30_BrNO_13_Na [M+Na]^+^ 678.0798, obs. 678.0785.

*N*-Acetyl-5-bromoindol-3-yl 2,3,4,5,7-penta-O-acetyl-β-d-*glycero*-d-*ido*-septanoside (**18**). Obtained as yellow solid from **17** using the general procedure (70% yield over 3 steps) R*_f_* 0.2 (3:2 Hex: EtOAc) ^1^H NMR (400 MHz, CD_3_OD) δ 8.20 (d, J = 8.8 Hz, 1H), 7.62 (d, J = 1.9 Hz, 1H), 7.41 (dd, J = 8.8, 1.9 Hz, 1H), 7.37 (broad s, 1H), 5.64 (d, J = 1.5 Hz, 1H), 5.59–5.55 (m, 2H), 5.49–5.45 (m, 1H), 5.36 (app t, J = 9.3 Hz, 1H) 4.33 (ddd, J = 9.2, 7.4, 3.6Hz, 1H), 4.23 (app d, J = 3.6 Hz, 2H), 2.60 (s, 3H), 2.15 (s, 3H), 2.06 (s, 3H), 2.02 (s, 3H), 2.00 (s, 3H), 1.76 (s, 3H); ^13^C NMR (100 MHz, CD_3_OD) δ 172.1, 171.3, 171.0, 170.9, 170.7, 170.6, 140.9, 133.4, 129.8, 127.3, 121.5, 119.0, 117.7, 114.9, 112.2, 101.6, 76.7, 73.2, 72.7, 71.3, 71.2, 64.4, 23.9, 20.6(2), 20.5; HRMS (DART-TOF) *m/z* calcd. for C_27_H_30_BrNO_13_Na [M+Na]^+^ 678.0798, obs. 678.0816.

*Zemplén De-acetylation (General Procedure).* A solution of the starting material (1.00 mmol) in MeOH (15.0–20.0 mL) was treated with a catalytic amount of sodium methoxide (5 mol%). The solution was stirred at room temperature overnight (~16 h). If the product had precipitated during this time it was collected by filtration, otherwise the solution was neutralized with Amberlite IR-120 (H+) resin (pH 7 by pH paper) and then the solution was concentrated to near dryness. The residue was then re-dissolved in a minimum amount of water (≤5.0 mL) and subjected to lyophilization.

5-Bromoindol-3-yl β-d-*glycero*-d-*gulo*-septanoside (**8**). Obtained from **14** as tan powder after lyophilization using the general procedure (75% yield). R*_f_* 0.4 (4:1 DCM: MeOH) ^1^H NMR (400 MHz, CD_3_OD) δ 7.75 (s, 1H), 7.23 (s, 1H), 7.19 (d, *J =* 8.7 Hz, 1H), 7.14 (d, *J =* 8.7 Hz, 1H), 4.99 (s, 6H), 4.93 (d, *J =* 5.5 Hz, 1H), 4.21 (app t, *J =* 4.4 Hz, 1H), 3.89 (dd, *J =* 9.1, 3.3 Hz, 1H), 3.86–3.79 (m, 2H), 3.66–3.60 (m, 2H), 3.46 (app t, *J =* 7.9 Hz, 1H); ^13^C NMR (100 MHz, CD3OD) δ 137.9, 133.9, 125.6, 123.2, 121.0, 114.2(2), 112.7, 110.1, 82.3, 75.6, 75.5, 73.9, 72.2, 64.4; HRMS (DART-TOF) *m/z* calcd. for C_15_H_19_BrNO_7_ [M+H]^+^ 404.0345, obs. 404.0338.

5-Bromoindol-3-yl β-d-*glycero*-d-*ido*-septanoside (**9**). Obtained as light brown solid from **18** using the general procedure (79% yield) R*_f_* 0.4 (4:1 DCM: MeOH) ^1^H NMR (400 MHz, CD_3_OD) δ 7.87 (s, 1H), 7.78 (d, *J =* 1.8 Hz, 1H), 7.20 (m, 1H), 7.16 (dd, *J =* 8.6, 1.8 Hz, 1H), 5.09 (d, *J =* 2.3 Hz, 1H), 4.88 (s, 6H), 4.00 (dd, *J =* 6.3, 2.2 Hz, 1H), 3.86–3.83 (m, 1H), 3.71–3.66 (m, 2H), 3.61 (dd, *J =* 14.1, 7.0 Hz, 1H), 3.52–3.50 (m, 2H); ^13^C NMR (100 MHz, CD_3_OD) δ 137.7, 133.9, 125.7, 123.3, 121.1, 114.2(2), 112.8, 105.5, 83.2, 76.7, 75.7, 75.5, 74.1, 64.3; HRMS (DART-TOF) *m/z* calcd. for C_15_H_18_BrNO_7_Na [M+Na]^+^ 426.0164, obs. 426.0204.

### 4.3. Synthesis of BODIPY-Labeled Septanoside ***23***

BODIPY:2,3,4,5,7-Penta-*O*-acetyl-β-d-*glycero*-d-*gulo*-septanosyl azide conjugate (**22**). BODIPY-FL alkyne **20** (0.0025 g, 0.008 mmol) and septanosyl azide **21** (0.0085 g, 0.02 mmol) and were dissolved in a mixture of THF:water (1 mL of 3:1 mixture). A solution of CuSO_4_·5H_2_O (0.001 g, 0.5 eq.) and sodium ascorbate (0.0012 g, 0.8 eq.) in an additional 1 mL of the THF:water was sonicated for 10 min and then added to the initial alkyne-azide solution. The mixture was allowed to react at room temperature with stirring for 12 h. After, the reaction mixture was diluted with 5 mL EtOAc and the aqueous and organic layers separated; the aqueous layer was then extracted with additional EtOAc (2 × 5 mL). The combined organic layers were dried over Na_2_SO_4_, filtered, and the solvent was evaporated under reduced pressure. The crude product was then purified by column chromatography to deliver **20** as brownish oil (0.0028 g, 48% yield) R*_f_* 0.4 (3:2 Hex: EtOAc); ^1^H NMR (400 MHz, CDCl_3_) δ 7.09 (s, 1H), 6.88 (d, *J =* 3.9 Hz, 1H), 6.28 (d, *J =* 4.0 Hz, 1H), 6.12 (s, 1H), 5.82 (broad s, 1H), 5.40 (dd, *J =* 8.0, 1.9 Hz, 2H), 5.34 (dd, *J =* 8.0, 3.9 Hz, 2H), 5.10 (dd, *J =* 6.5, 1.9 Hz, 2H), 5.05–5.00 (m, 2H), 4.18–4.12 (m, 3H), 3.28 (t, *J =* 7.5 Hz, 2H), 2.67–2.63 (m, 2H), 2.57 (s, 3H), 2.26 (s, 3H), 2.12 (s, 3H), 2.11 (s, 9H), 2.09 (s, 3H); ^13^C NMR (100 MHz, CDCl_3_) δ 171.5, 170.8, 169.3, 169.1 (2), 128.5, 124.0, 120.7, 117.7, 91.3, 79.7, 77.4, 76.0, 72.1, 71.8, 71.6, 70.6, 69.4, 64.3, 36.1, 29.9, 29.4, 25.0, 20.9 (3), 15.2, 11.5;HRMS (DART-TOF) *m/z* calcd. for C_34_H_41_BF_2_N_6_O_12_Na [M+Na]^+^ 797.2730, obs. 797.2725.

BODIPY: β-d-*glycero*-d-gulo-septanosyl azide conjugate (**23**). A solution of starting per-*O*-acetylated BODIPY conjugate **22** (0.0028 g, 0.004 mmol) was dissolved in dry methanol (0.75 mL) and kept under an inert atmosphere and dark conditions. To this solution was added 5 mol % of NaOMe in MeOH. The reaction was stirred at room temperature for 12 h and monitored by TLC (R*_f_* of the product is 0.1 and starting material is 0.5 in 1:1 Hex/EtOAc). Upon complete disappearance of the starting material, the reaction solvent was evaporated under the reduced pressure and the residue was re-dissolved in de-ionized water (1.00 mL) and subjected to lyophilization to obtain **23** as a brownish solid. The crude product was used in confocal microscopy experiments without further purification.

### 4.4. Glycosidase Activity Assay on E. coli Cells

Twenty microliters of 100 mM IPTG solution in water and 20 μL of a 20 mg/mL X-Gal (5) or X-Sept (**8**) solution in DMF were spread on LB agar Petri plates (20 mL medium) containing kanamycin (100 μg/mL) and allowed to dry for 20 min at 37 °C. Competent BL21 (DE3) Star cells were transformed (chemically competent cells [37]) with plasmid pRSF-β-gal encoding the *E. coli* β-galactosidase and spread onto the agar plates. Color development was analyzed after 24 h incubation at 37 °C.

### 4.5. Probing Glycoside Hydrolases for Septanoside Hydrolysis

Activity assays were performed at the optimal pH and temperature for each enzyme as indicated in Table 1. Indolyl substrates **8** and **9** (1 mM) and enzymes (concentration ranging from 25 to 50 U/mL) in buffer were incubated in 1 mL cuvettes and the absorbance monitored at 630 nm. Specific activities of the commercial enzymes with pNP substrates are from the manufacturer, and β-glucosidase Bgl3 from *Streptomyces* sp. as reported [30].

### 4.6. Kinetics of Streptomyces β-Glucosidase with p-Nitrophenyl Septanoside ***19***

Kinetics were performed by monitoring *p*NP release by absorbance at 400 nm. Reactions were done in thermostated cuvettes at 50 °C in 50 mM phosphate buffer pH 6.5 with 0.1–10 mM substrates and 46 nM enzyme (Bgl3) for *p*NP-Glc or 9.2 μM Bgl3 for *p*NP-septanoside (**19**). Rates were obtained from the initial slopes after subtracting the blank rates (in the absence of enzyme) and kinetic constants were calculated from data fitted to a Michaelis–Menten equation with substrate inhibition (Equation (1)) for *p*NP-Glc and to a sigmoidal equation (Equation (2)) for *p*NP-Sept (**19**) using GraphPad software (Prism, San Diego, CA, USA).

(1) $$v=k_{cat}{\left[E\right]}_{0}\left[S\right]/(K_{M}+\left[S\right]+{\left[S\right]}^{2}/K_{i})$$

(2) $$v=k_{cat}{\left[E\right]}_{0}{\left[S\right]}^{h}/\left(K_{M}^{\;\;\;h}+{\left[S\right]}^{h}\right)$$

where [*S*] is the substrate concentration, [*E*]_0_ the enzyme concentration, and *k_cat_* (catalytic constant), *K_M_* (Michaelis constant), *K_i_* (substrate inhibition constant) and h (Hill index) are the adjustable parameters [38].

### 4.7. Cell Internalization of Compound ***23*** by Confocal Microscopy

*E. coli* MG1655 cultures were grown to an OD_600_ of ~1.0 (1 × 10^9^ cfu/mL) in Luria Broth (LB), washed, and suspended in 10 mM MES buffer pH 5.5 to a final OD_600_ = 1. To 100 μL of cell suspension (different dilutions tested, best results with a 1:2 dilution), BODIPY-septanoside **23** was added to a final 1 μM concentration with shaking. Cells were incubated for 1 h at 37 °C. Then, 1 μL FM 4–64 (1 μg/mL stock) and 1 μL DAPI (2 μg/mL stock) were added to the cells with shaking and incubated for 30 min at 37 °C for membrane and DNA staining, respectively. Cells were spun down by centrifugation for 60 s and resuspended in 1/10 their original volume. Three microliters of cell suspension were spotted onto 1.5% agarose pads containing solid LB medium. The cells were then imaged using a Nikon A1R spectral confocal microscope with a 60× oil immersion lens.

## Figures and Tables

**Table 1:** In vitro screen of selected glycosidases.

Enzyme	Substrate	S.A. (U/mg) ^a^	Conditions
β-Galactosidase (*Escherichia coli*)	pNP-Gal	35	Phosphate buffer (100 mM) pH 6.5 40 °C
	**8**	n.d. ^b^	
	**9**	n.d.	
β-Galactosidase (*Aspergillus niger*)	pNP-Gal	170	Acetate buffer (100 mM) pH 4.5 40 °C
	**8**	<0.001	
	**9**	n.d.	
β-Glucosidase (*Streptomyces* sp.)	pNP-Glc	3.3	Phosphate buffer (50 mM) pH 6.5 50 °C
	**8**	<0.005	
	**9**	n.d.	
β-Glucosidase (*Almonds*)	pNP-Glc	2	Phosphate buffer (100 mM) pH 5.0 37 °C
	**8**	n.d.	
	**9**	n.d.	
β-Glucosidase (*Thermatoga maritima*)	pNP-Glc	70	Maleate buffer (50 mM) pH 6.5 40 °C
	**8**	n.d.	
	**9**	n.d.	
β-Glucosidase (*Phanerochaete chyrosporium*)	pNP-Glc	100	Acetate buffer (100 mM) pH 5.0 40 °C
	**8**	n.d.	
	**9**	n.d.	
β-Mannosidase (*Cellulomonas fimi*)	pNP-Man	10	Maleate buffer (100 mM) pH 6.5 35 °C
	**8**	<0.001	
	**9**	<0.001	
β-Glucuronidase (*Escherichia coli*)	pNP-GlcA	110	Tris·HCl buffer (100 mM) pH 7.5 37 °C
	**8**	n.d.	
	**9**	n.d.	
β-Xylosidase (*Bacillus pumilus*)	pNP-Xyl	18	Phosphate buffer (50 mM) pH 7.5 35 °C
	**8**	n.d.	
	**9**	n.d.	

^a^ Specific activity (U/mg) at 1 mM pNP-substrates. ^b^ n.d., not detected.

## Data Availability

The data that support the findings of this study are available from the corresponding authors upon reasonable request.

## Supplementary Materials

The following are available online at https://www.mdpi.com/article/10.3390/ijms22094497/s1.

## References

[1] Pallister E., Gray C.J., Flitsch S.L. (2020). Enzyme promiscuity of carbohydrate active enzymes and their applications in biocatalysis. Curr. Opin. Struct. Biol..

[2] Kytidou K., Artola M., Overkleeft H.S., Aerts J.M.F.G. (2020). Plant Glycosides and Glycosidases: A Treasure-Trove for Therapeutics. Front. Plant Sci..

[3] Gloster T.M. (2020). Exploitation of carbohydrate processing enzymes in biocatalysis. Curr. Opin. Chem. Biol..

[4] Davies G.J., Planas A., Rovira C. (2012). Conformational Analyses of the Reaction Coordinate of Glycosidases. Acc. Chem. Res..

[5] Sinnott M.L. (1990). Catalytic Mechanisms of Enzymic Glycosyl Transfer. Chem. Rev..

[6] Lombard V., Golaconda Ramulu H., Drula E., Coutinho P.M., Henrissat B. (2014). The carbohydrate-active enzymes database (CAZy) in 2013. Nucleic Acids Res..

[7] Dey S., Samanta G.C., Jayaraman N., Rauter A.P., Christensen B., Somsak L., Kosma P., Adamo R. (2020). Advancements in synthetic and structural studies of septanoside sugars. Recent Trends in Carbohydrate Chemistry.

[8] Saha J., Peczuh M.W. (2011). Synthesis and properties of septanose carbohydrates. Advances in Carbohydrate Chemistry and Biochemistry.

[9] Ben Nejma A., Nguir A., Ben Jannet H., Hamza M.A., Daïch A., Othman M., Lawson A.M. (2015). New septanoside and 20-hydroxyecdysone septanoside derivative from Atriplex portulacoides roots with preliminary biological activities. Bioorg. Med. Chem. Lett..

[10] Le Merrer Y., Poitout L., Depezay J.C., Dosbaa I., Geoffroy S., Foglietti M.J. (1997). Synthesis of azasugars as potent inhibitors of glycosidases. Bioorg. Med. Chem..

[11] Tauss A., Steiner A.J., Stütz A.E., Tarling C.A., Withers S.G., Wrodnigg T.M. (2006). l-Idoseptanosides: Substrates of d-glucosidases?. Tetrahedron Asymmetry.

[12] Elshahawi S.I., Shaaban K.A., Kharel M.K., Thorson J.S. (2015). A comprehensive review of glycosylated bacterial natural products. Chem. Soc. Rev..

[13] Guo Z., Tang Y., Tang W., Chen Y. (2021). Heptose-containing bacterial natural products: Structures, bioactivities, and biosyntheses. Nat. Prod. Rep..

[14] Tang W., Guo Z., Cao Z., Wang M., Li P., Meng X., Zhao X., Xie Z., Wang W., Zhou A. (2018). D-Sedoheptulose-7-phosphate is a common precursor for the heptoses of septacidin and hygromycin B. Proc. Natl. Acad. Sci. USA.

[15] Horwitz J.P., Chua J., Cubby R.J., Tomson A.J., Da Rooge M.A., Fisher B.E., Mauricio J., Klundt I. (1964). Substrates for Cytochemical Demonstration of Enzyme Activity. I. Some Substituted 3-Indolyl-β-D-glycopyranosides. J. Med. Chem..

[16] Miller J.H. (1972). Experiments in Molecular Genetics.

[17] Kiernan J. (2007). Indigogenic substrates for detection and localization of enzymes. Biotech. Histochem..

[18] Böttcher S., Thiem J. (2014). Indoxylic Acid Esters as Convenient Intermediates Towards Indoxyl Glycosides. Eur. J. Org. Chem..

[19] Wolk J.L., Frimer A.A. (2010). A simple, safe and efficient synthesis of Tyrian purple (6,6’-Dibromoindigo). Molecules.

[20] Castro S., Duff M., Snyder N.L., Morton M., Kumar C.V., Peczuh M.W. (2005). Recognition of septanose carbohydrates by concanavalin A. Org. Biomol. Chem..

[21] Duff M.R., Fyvie W.S., Markad S.D., Frankel A.E., Kumar C.V., Gascón J.A., Peczuh M.W. (2011). Computational and experimental investigations of mono-septanoside binding by Concanavalin A: Correlation of ligand stereochemistry to enthalpies of binding. Org. Biomol. Chem..

[22] Sager C.P., Fiege B., Zihlmann P., Vannam R., Rabbani S., Jakob R.P., Preston R.C., Zalewski A., Maier T., Peczuh M.W. (2018). The price of flexibility—a case study on septanoses as pyranose mimetics. Chem. Sci..

[23] Nagata S., Tomida H., Iwai-Hirose H., Tanaka H.N., Ando H., Imamura A., Ishida H. (2019). Synthesis of a 1,2-: Cis -indoxyl galactoside as a chromogenic glycosidase substrate. RSC Adv..

[24] Böttcher S., Thiem J. (2015). Synthesis of indoxyl-glycosides for detection of glycosidase activities. J. Vis. Exp..

[25] Böttcher S., Hederos M., Champion E., Dékány G., Thiem J. (2013). Novel efficient routes to indoxyl glycosides for monitoring glycosidase activities. Org. Lett..

[26] Pote A.R., Vannam R., Richard A., Gascón J., Peczuh M.W. (2018). Formation of and Glycosylation with Per-O-Acetyl Septanosyl Halides: Rationalizing Complex Reactivity En Route to p-Nitrophenyl Septanosides. Eur. J. Org. Chem..

[27] de Melo J.S.S., Rondão R., Burrows H.D., Melo M.J., Navaratnam S., Edge R., Voss G. (2006). Spectral and Photophysical Studies of Substituted Indigo Derivatives in Their Keto Forms. ChemPhysChem.

[28] Rajesh K., Somasundaram M., Saiganesh R., Balasubramanian K.K. (2007). Bromination of deactivated aromatics: A simple and efficient method. J. Org. Chem..

[29] Imming P., Imhof I., Zentgraf M. (2001). An improved synthetic procedure for 6,6′-dibromoindigo (Tyrian Purple). Synth. Commun..

[30] Vallmitjana M., Ferrer-Navarro M., Planell R., Abel M., Ausín C., Querol E., Planas A., Pérez-Pons J.A. (2001). Mechanism of the family 1 β-glucosidase from Streptomyces sp: Catalytic residues and kinetic studies. Biochemistry.

[31] Stroobants A., Portetelle D., Vandenbol M. (2014). New carbohydrate-active enzymes identified by screening two metagenomic libraries derived from the soil of a winter wheat field. J. Appl. Microbiol..

[32] Kowada T., Maeda H., Kikuchi K. (2015). BODIPY-based probes for the fluorescence imaging of biomolecules in living cells. Chem. Soc. Rev..

[33] Uppal T., Bhupathiraju N.V.S.D.K., Vicente M.G.H. (2013). Synthesis and cellular properties of Near-IR BODIPY-PEG and carbohydrate conjugates. Tetrahedron.

[34] Aharoni A., Thieme K., Chiu C.P.C., Buchini S., Lairson L.L., Chen H., Strynadka N.C.J., Wakarchuk W.W., Withers S.G. (2006). High-throughput screening methodology for the directed evolution of glycosyltransferases. Nat. Methods.

[35] Liu B., Novikova N., Simpson M.C., Timmer M.S.M., Stocker B.L., Söhnel T., Ware D.C., Brothers P.J. (2016). Lighting up sugars: Fluorescent BODIPY-: Gluco -furanose and -septanose conjugates linked by direct B-O-C bonds. Org. Biomol. Chem..

[36] Kolb H.C., Finn M.G., Sharpless K.B. (2001). Click Chemistry: Diverse Chemical Function from a Few Good Reactions. Angew. Chem. Int. Ed..

[37] Green M.R., Hughes H., Sambrook J., MacCallum P. (2012). Molecular Cloning; A Laboratory Manual.

[38] Segel I.H. (1975). Enzyme Kinetics: Behaviour and Analysis of Rapid Equilibrium and Steady-State Enzyme Systems.

