# Supplementary material for: Indolyl Septanoside Synthesis for In Vivo Screening of Bacterial Septanoside Hydrolases
Source: Int J Mol Sci. 2021 Apr 26;22(9):4497. doi: 10.3390/ijms22094497 (PMC8123396; doi:10.3390/ijms22094497)
Supplement: Supplementary file 1 [file ijms-22-04497-s001.zip › ijms-1184352-supplementary.pdf]

SUPPLEMENTARY MATERIALS

for

Indolyl-septanoside synthesis for *in vivo* screening of bacterial septanoside hydrolases

Aditya R. Pote[1], Sergi Pascual[2], Antoni Planas[2,*], Mark W. Peczuh[1,*]

[1] Department of Chemistry, University of Connecticut, 55 N. Eagleville Road U3060, Storrs, CT, 06279 USA.
[2] Laboratory of Biochemistry, Institut Químic de Sarrià, University Ramon Llull, 08017, Barcelona, Spain.

*Corresponding authors: antoni.planas@iqs.edu and mark.peczuh@uconn.edu

Contents

Item	Page
**Figure S1.** Relative fluorescence intensity from confocal scanning microscopy images (from Figure 6, main text)	2
**Figure S2.** Confocal fluorescence microscopy of *E. coli* TOP10 cells. DIC, optical microscopy image; Sept-Bodipy, BODIPY labeled septanoside (**23**). Lower resolution than with MG1655 cells (Figure 6, main text) due to smaller cells size.	2
**Figure S3.** UV-Vis spectrum of tyrian purple from independent synthesis (Tyrian Purple) and from hydrolysis of indolyl-septanoside **9** (precipitate).	3
NMR Spectra of new compounds:<br>Compounds: **13**, **17**, **14**, **18**, **8**, **9**, **22**	4-10

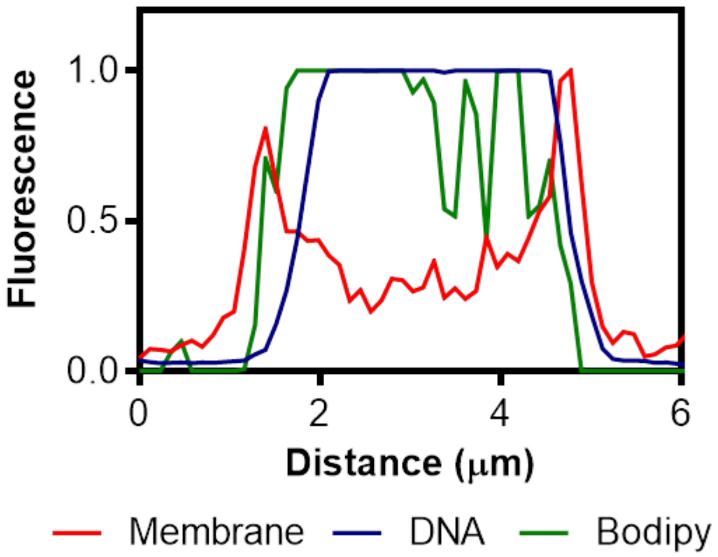

Figure S1. Relative fluorescence intensity from confocal scanning microscopy images (from Figure 6, main text)

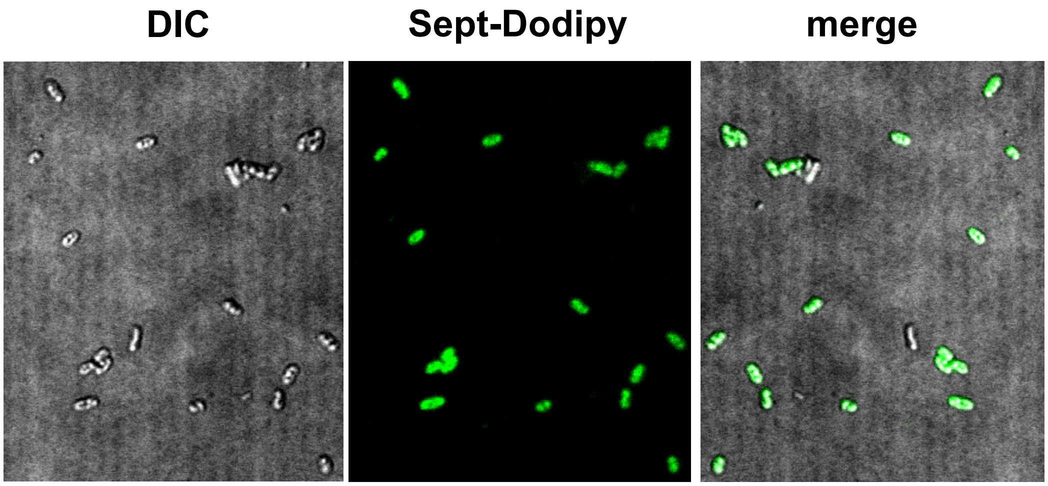

Figure S2. Confocal fluorescence microscopy of *E. coli* TOP10 cells. DIC, optical microscopy image; Sept-Bodipy, BODIPY-labelled septanoside (**23**). Lower resolution than with MG1655 cells (Figure 6, main text) due to smaller cells size.

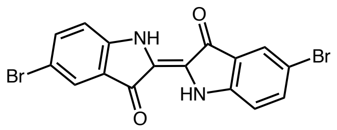

tyrian purple

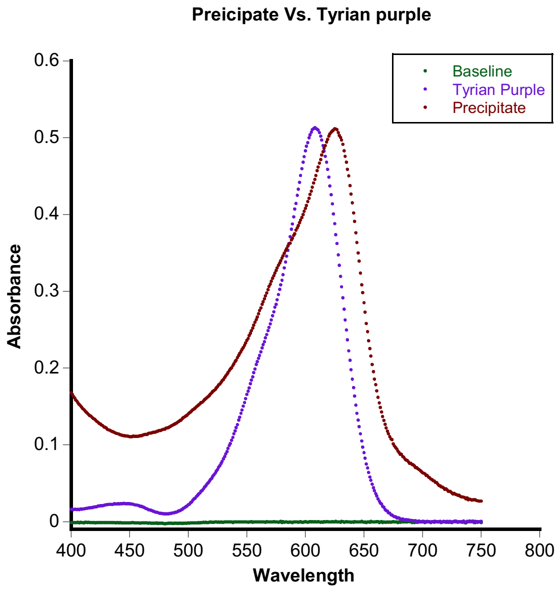

Figure S3. UV-Vis spectrum of tyrian purple from independent synthesis (Tyrian Purple) and from hydrolysis of indolyl-septanoside **9** (Precipitate).

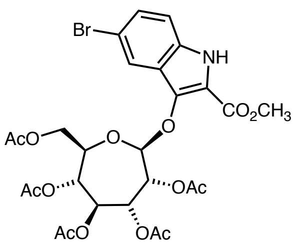

13

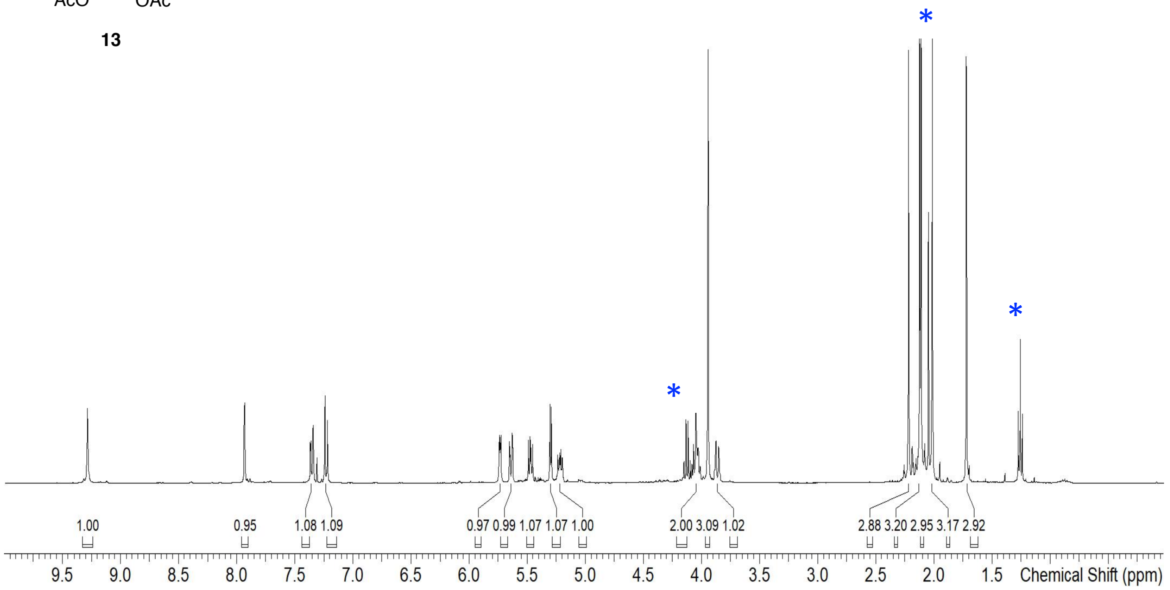

** indicates signals from residual ethyl acetate (EtOAc) in sample*

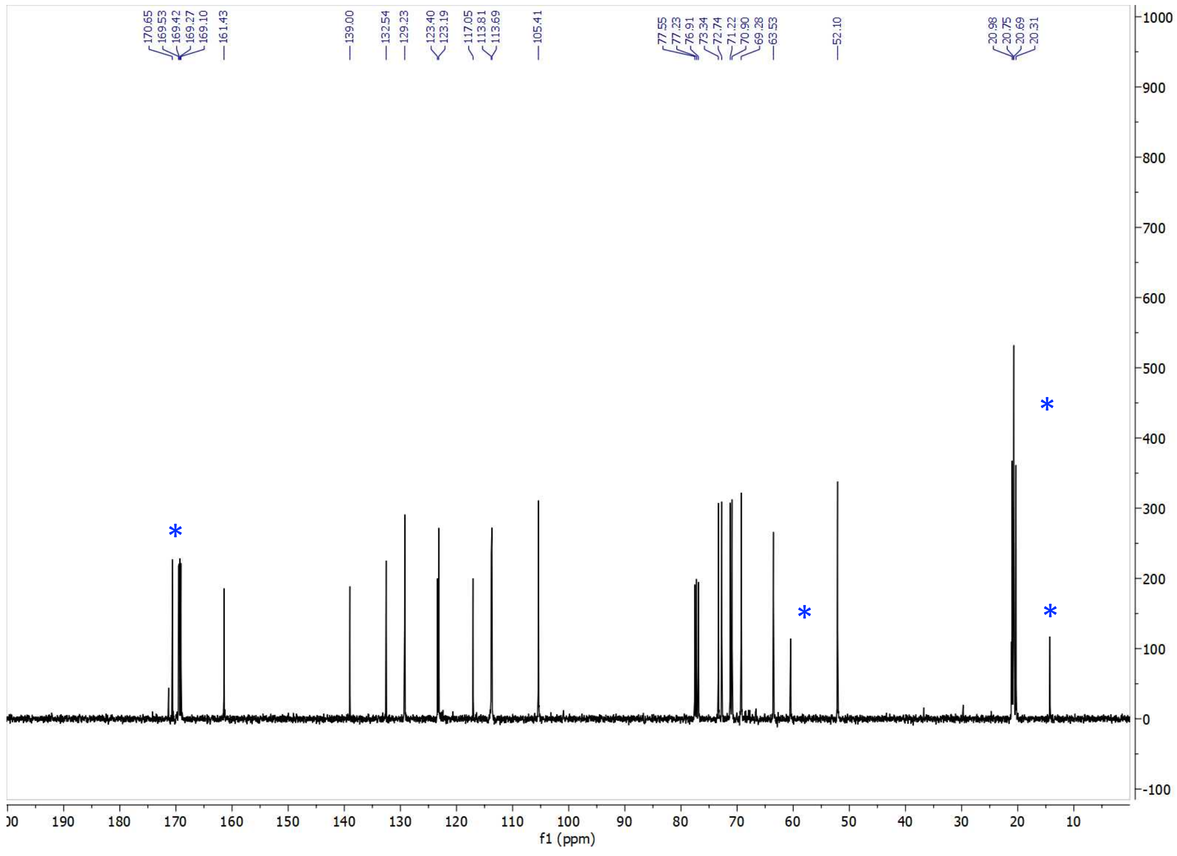

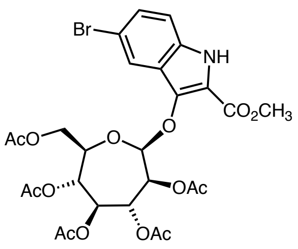

17

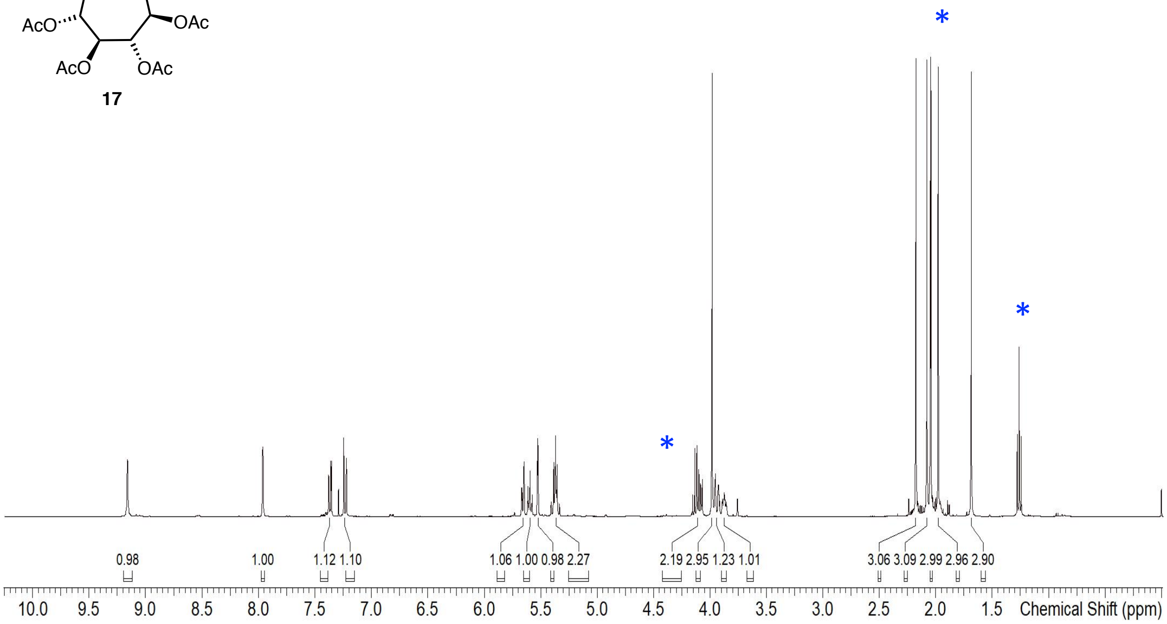

** indicates signals from residual ethyl acetate (EtOAc) in sample*

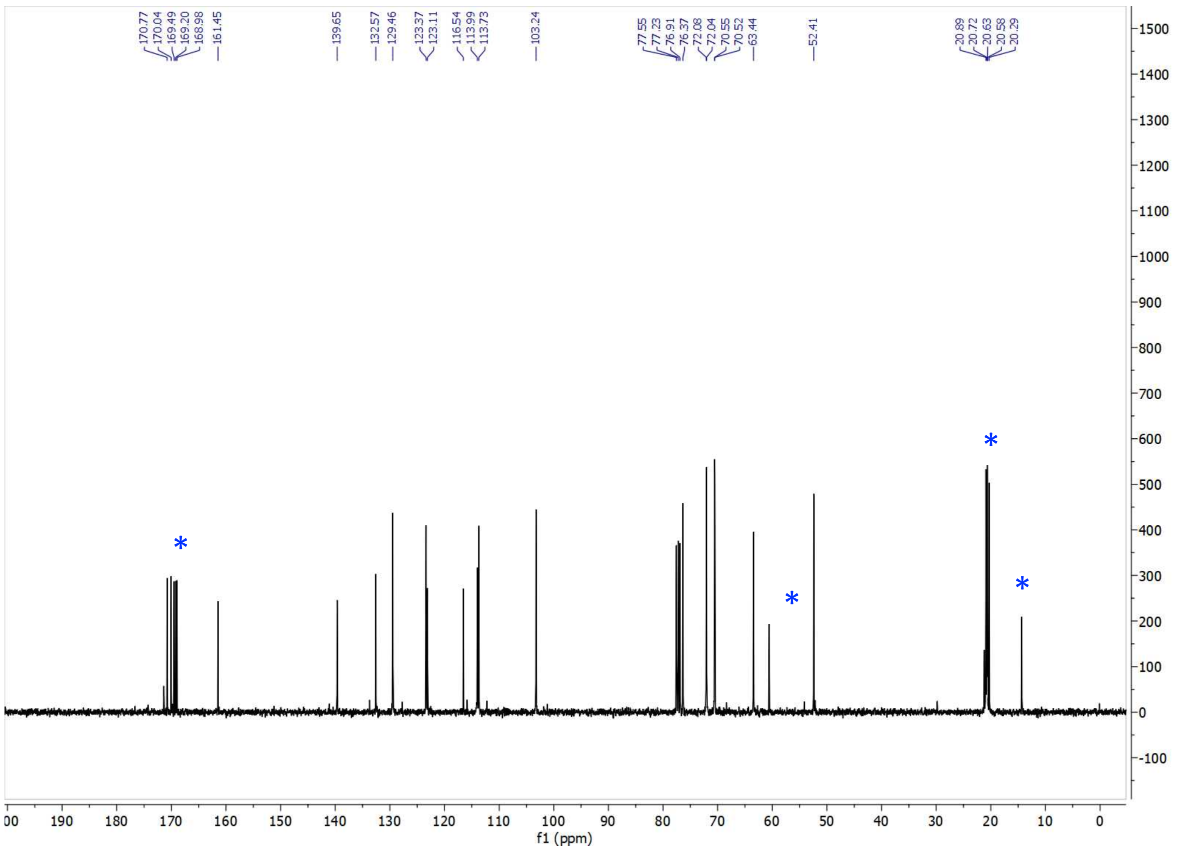

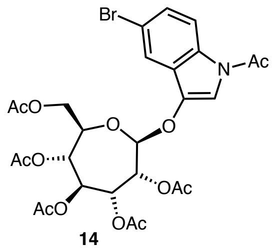

14

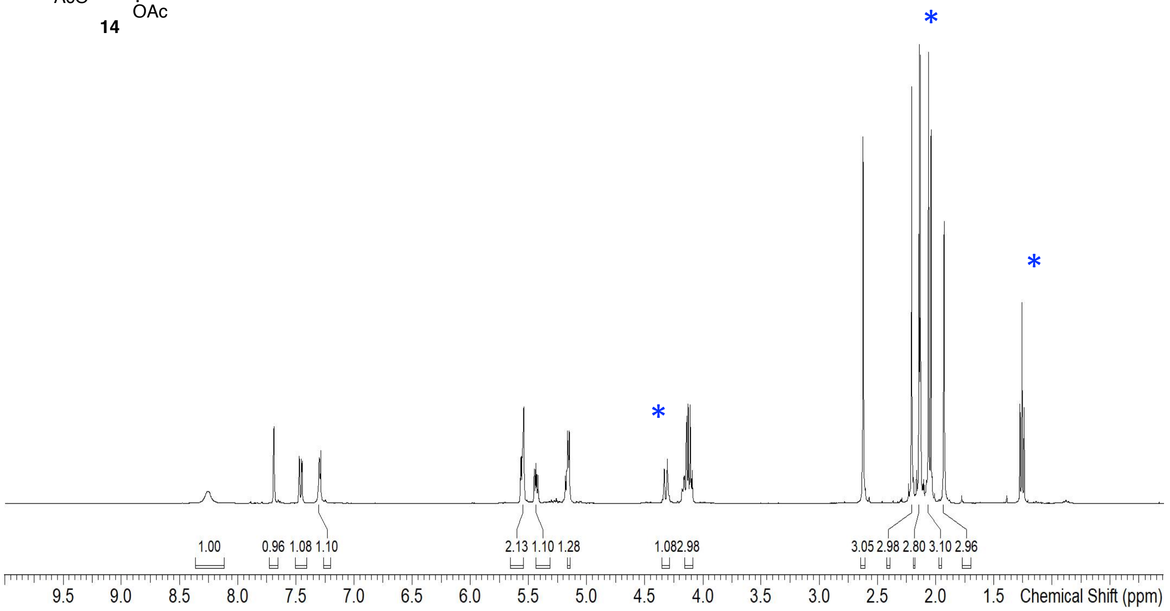

** indicates signals from residual ethyl acetate (EtOAc) in sample*

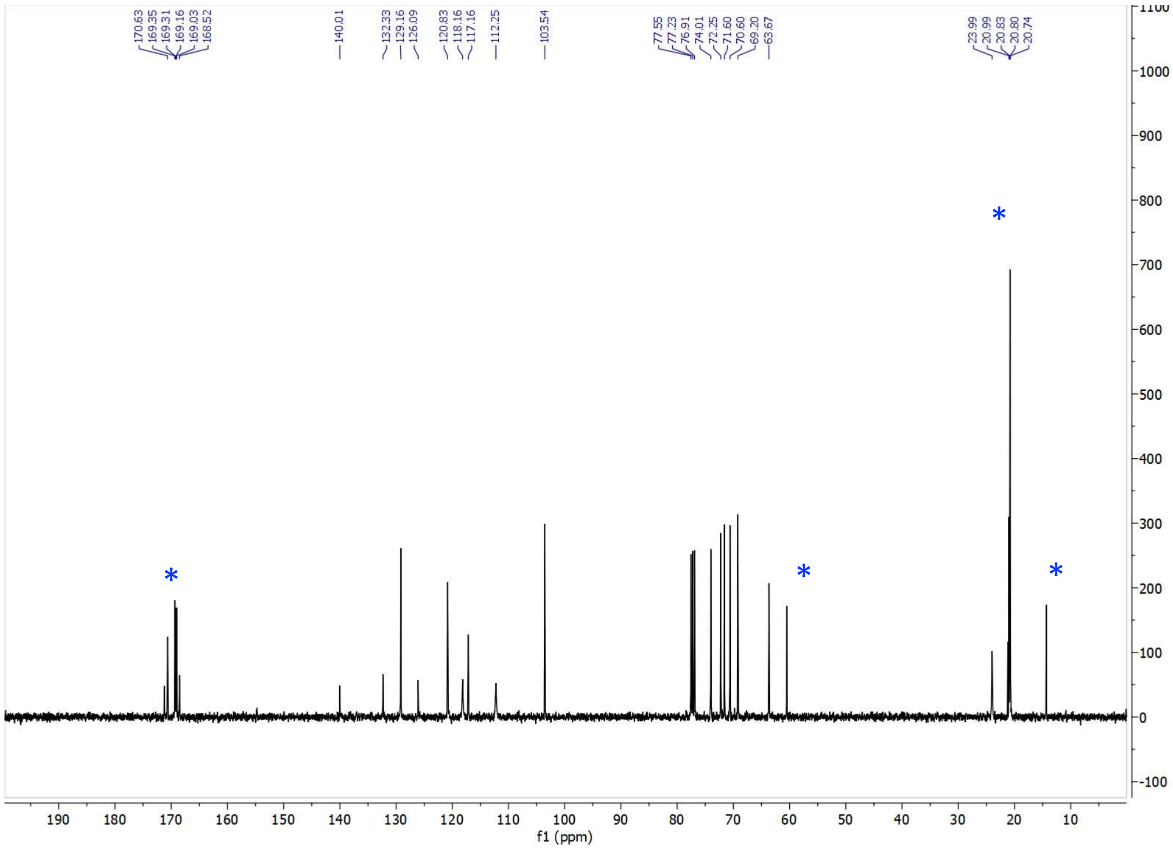

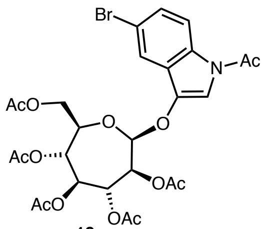
Br
N
Ac
AcO
AcO
AcO
O
O
OAc
OAc

18

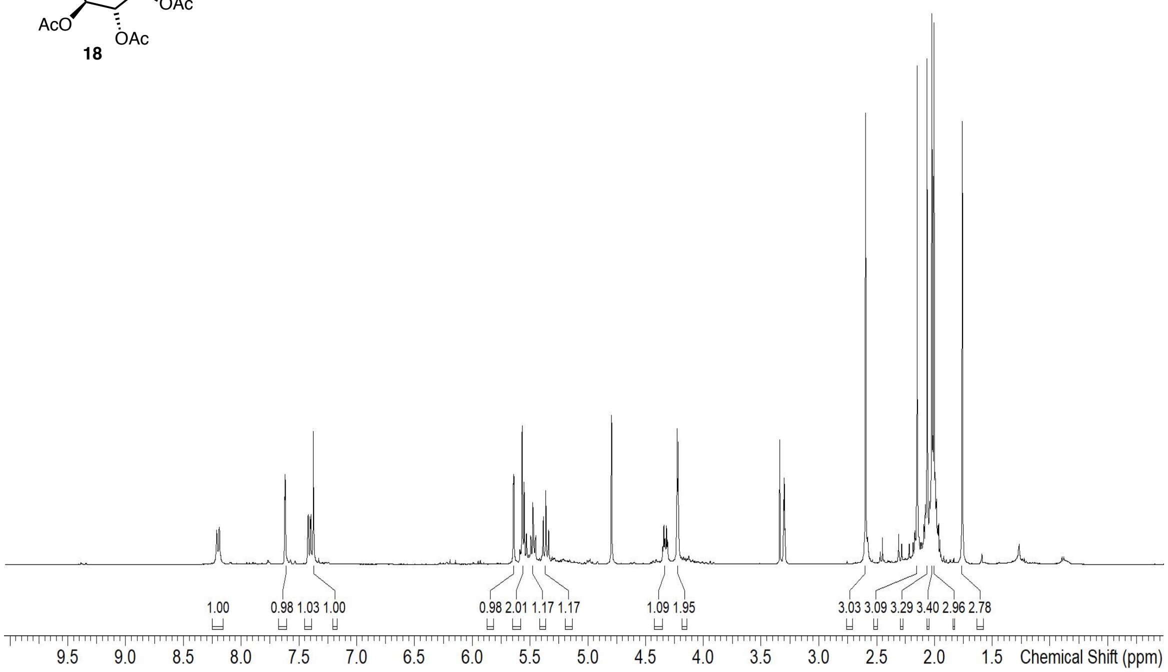
1.00
0.98 1.03 1.00
0.98 2.01 1.17 1.17
1.09 1.95
3.03 3.09 3.29 3.40 2.96 2.78
9.5 9.0 8.5 8.0 7.5 7.0 6.5 6.0 5.5 5.0 4.5 4.0 3.5 3.0 2.5 2.0 1.5
Chemical Shift (ppm)

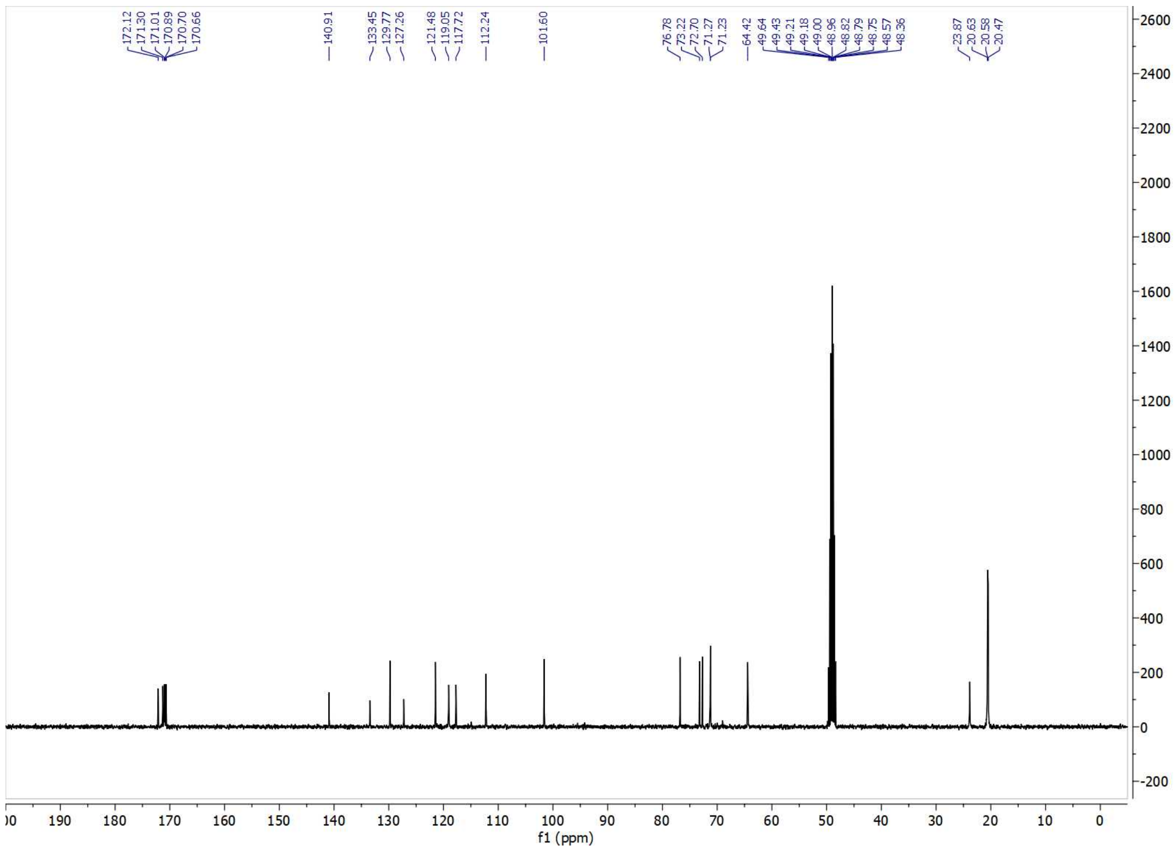
172.12
171.30
171.01
170.89
170.70
170.66
140.91
133.45
129.77
127.26
121.48
119.05
117.72
112.24
101.60
76.78
73.22
72.70
71.27
71.23
64.42
49.64
49.43
49.21
49.18
49.00
48.96
48.82
48.79
48.75
48.57
48.36
23.87
20.63
20.58
20.47
f1 (ppm)

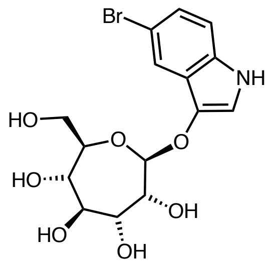
Br
NH
HO
O
O
HO
OH
HO
OH

8

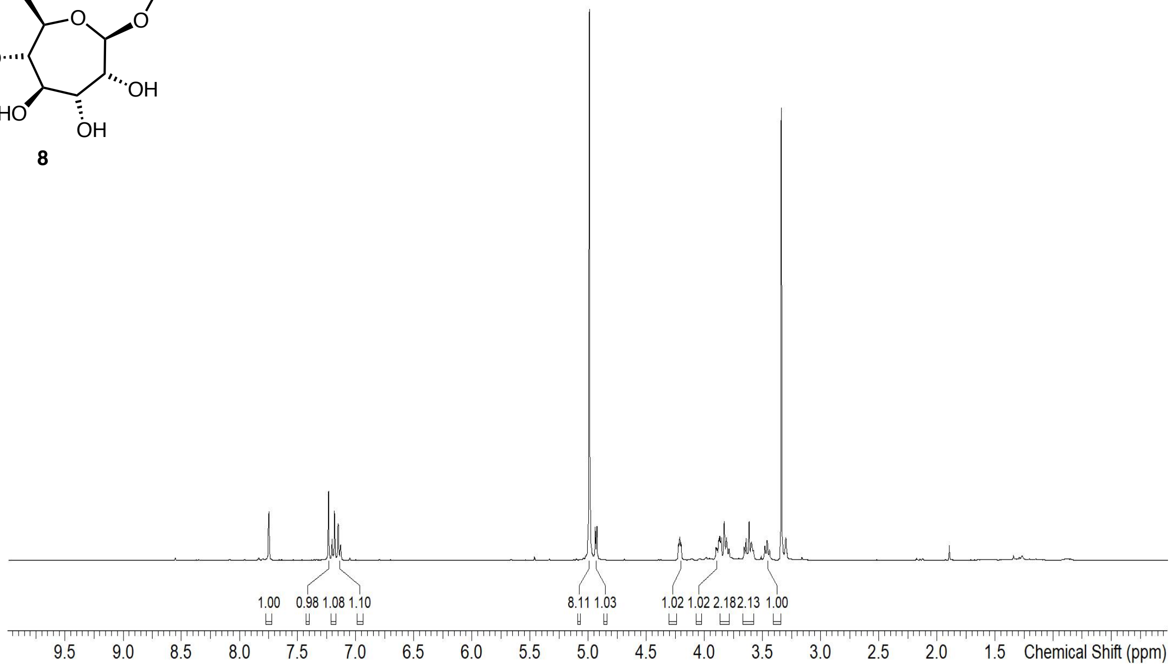
1.00 0.98 1.08 1.10
8.11 1.03
1.02 1.02 2.18 2.13 1.00
9.5 9.0 8.5 8.0 7.5 7.0 6.5 6.0 5.5 5.0 4.5 4.0 3.5 3.0 2.5 2.0 1.5 Chemical Shift (ppm)

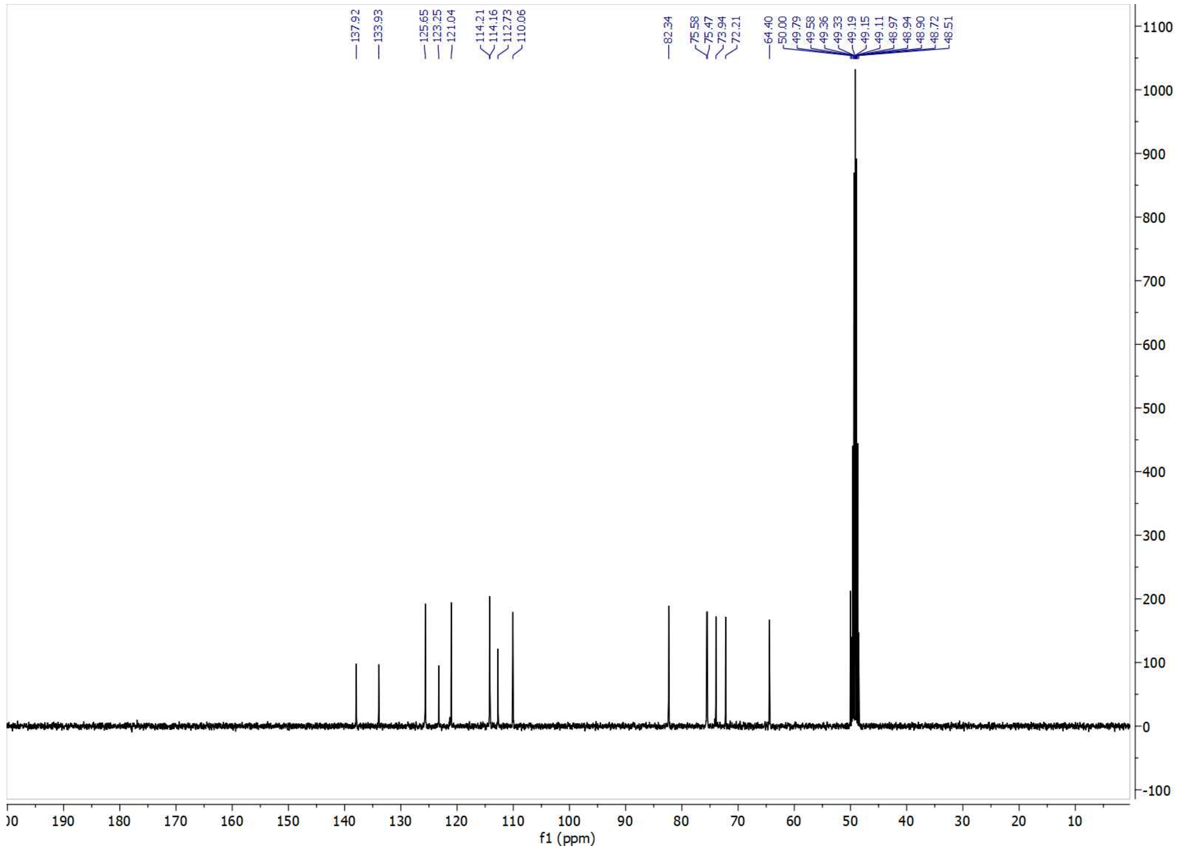
137.92
133.93
125.65
123.25
121.04
114.21
114.16
112.73
110.06
82.34
75.58
75.47
73.94
72.21
64.40
50.00
49.79
49.58
49.36
49.33
49.19
49.15
49.11
48.97
48.94
48.90
48.72
48.51
1100
1000
900
800
700
600
500
400
300
200
100
0
-100
190 180 170 160 150 140 130 120 110 100 90 80 70 60 50 40 30 20 10
f1 (ppm)

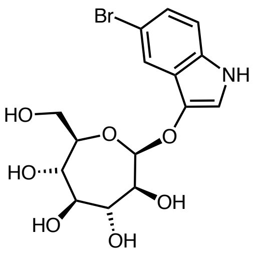
Br
NH
HO
O
O
HO
OH
HO
OH

9

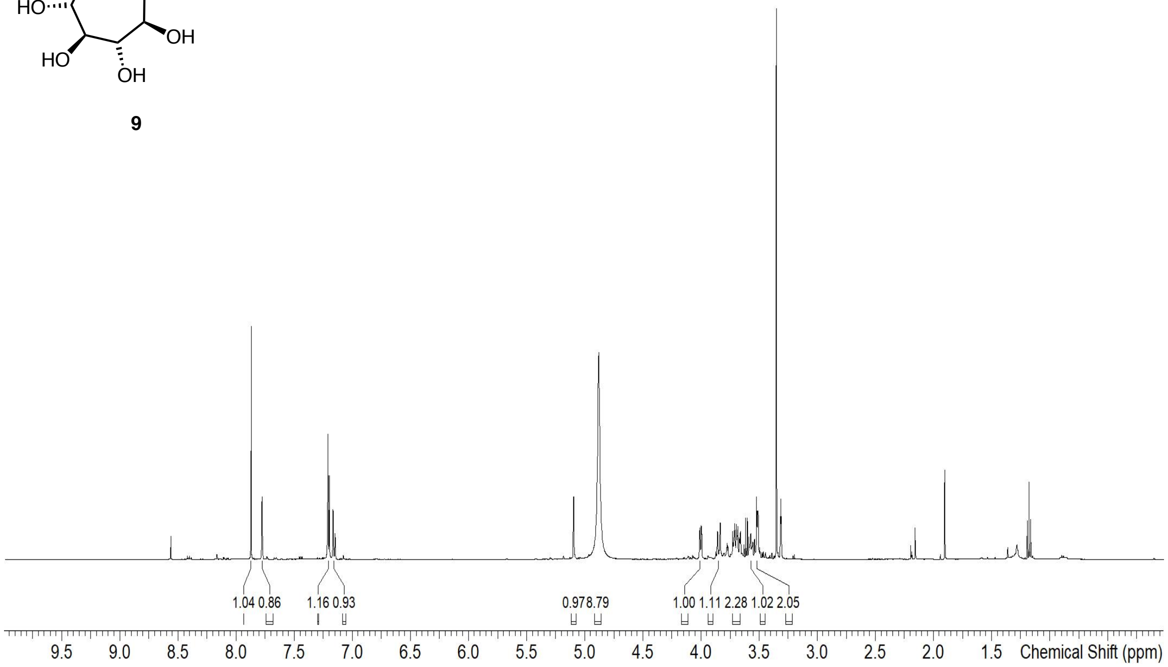
1.04 0.86
1.16 0.93
0.97 8.79
1.00 1.11 2.28 1.02 2.05
9.5 9.0 8.5 8.0 7.5 7.0 6.5 6.0 5.5 5.0 4.5 4.0 3.5 3.0 2.5 2.0 1.5
Chemical Shift (ppm)

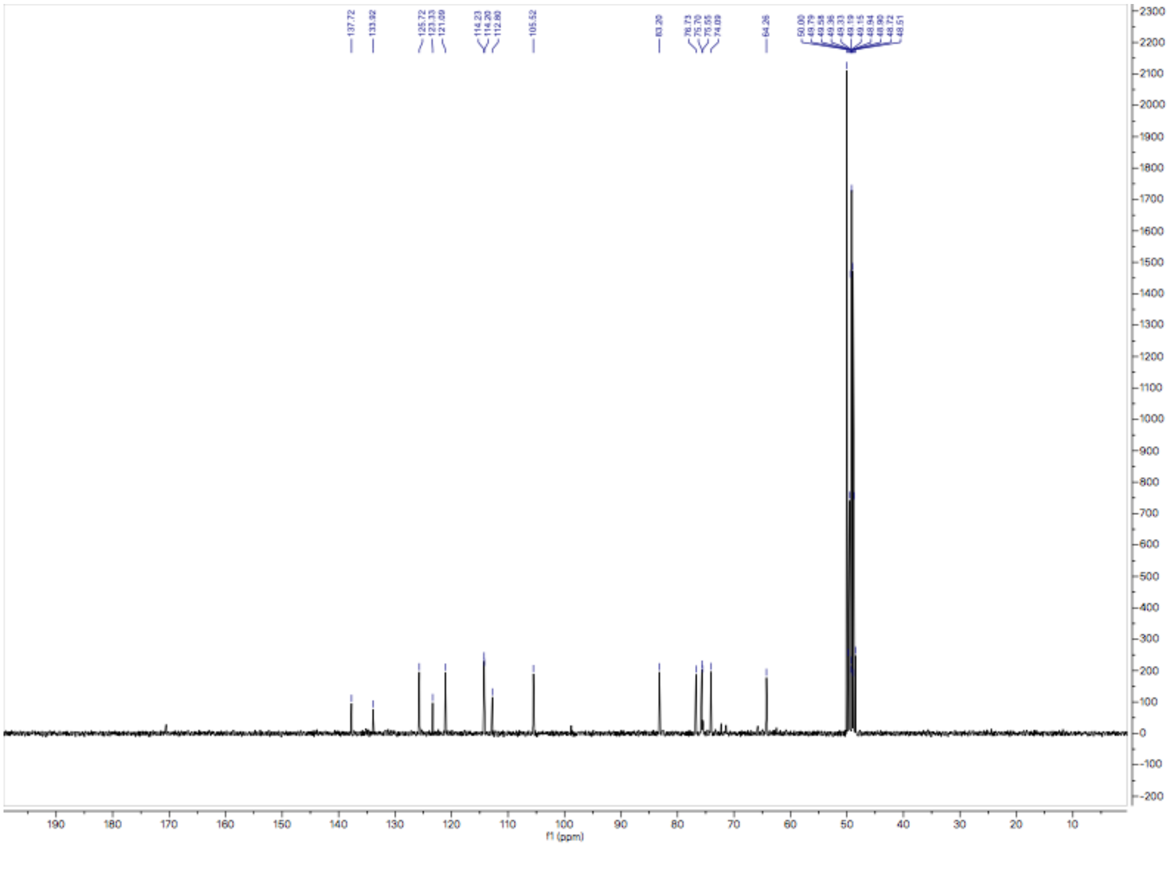
137.72
133.92
125.72
123.33
121.09
114.23
114.20
112.80
105.52
83.20
76.73
75.70
75.55
74.09
64.26
50.00
49.79
49.58
49.36
49.33
49.19
49.15
48.94
48.90
48.72
48.51
f1 (ppm)

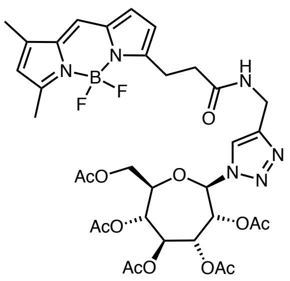
AcO
AcO
AcO
OAc
OAc
N
B
F
F
N
H
O
N
N
N
O

22

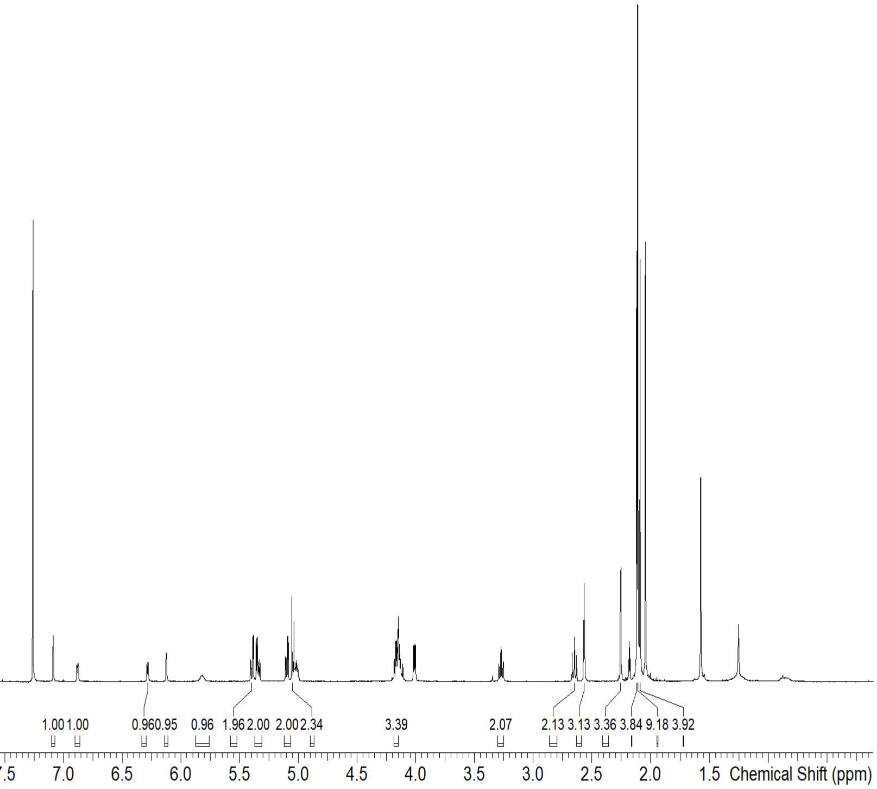
1.00
1.00
0.96
0.95
0.96
1.96
2.00
2.00
2.34
3.39
2.07
2.13
3.13
3.36
3.84
9.18
3.92
9.5
9.0
8.5
8.0
7.5
7.0
6.5
6.0
5.5
5.0
4.5
4.0
3.5
3.0
2.5
2.0
1.5
Chemical Shift (ppm)

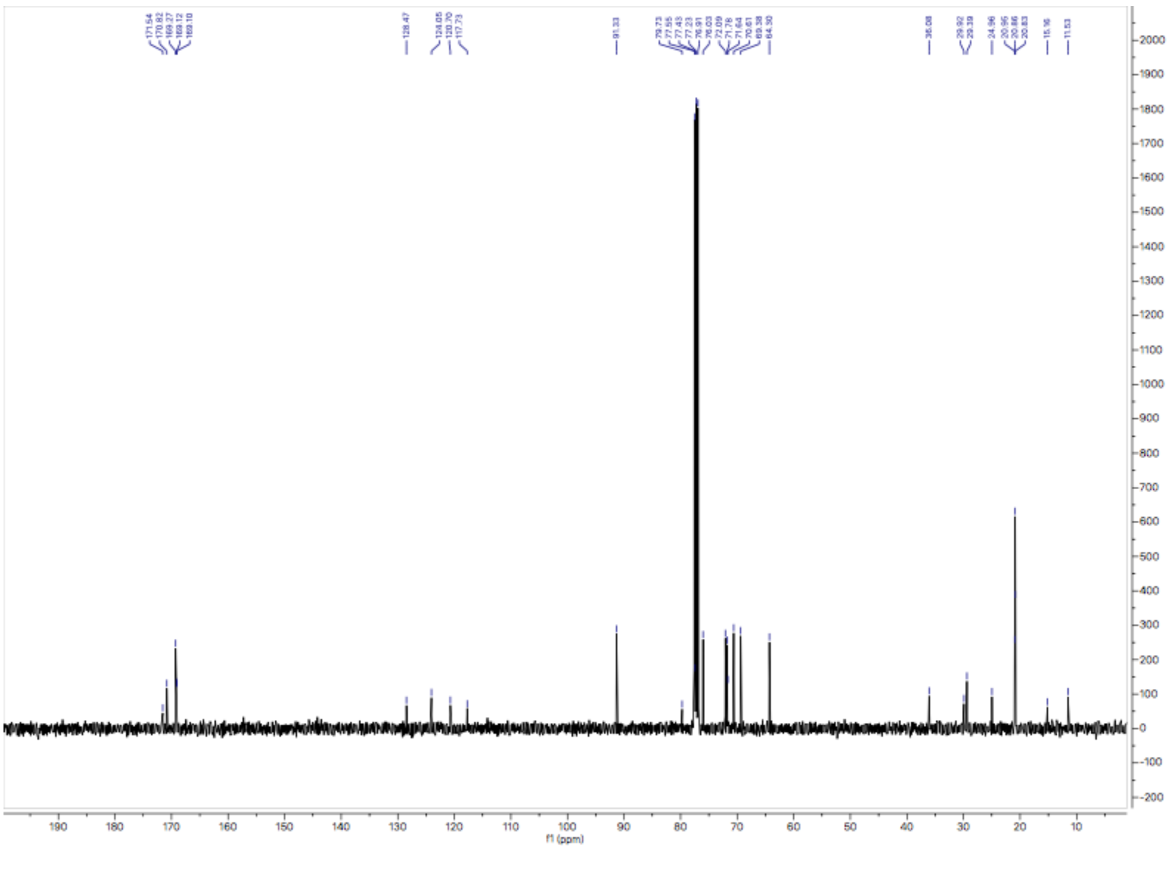
171.54
170.82
169.27
169.12
169.10
128.47
124.05
120.70
117.73
91.33
79.73
77.55
77.43
77.23
76.91
76.03
72.09
71.78
71.64
70.61
69.38
64.30
36.08
29.92
29.39
24.96
20.96
20.86
20.83
15.16
11.53
f1 (ppm)
